# Gate-Tunable Orbital Magnetism and Competing Superconductivity in Twisted Trilayer Graphene Josephson Junctions

**DOI:** 10.1021/acsami.5c15822

**Published:** 2025-12-08

**Authors:** Vishal Bhardwaj, Lekshmi Rajagopal, Lorenzo Arici, Matan Bocarsly, Alexey Ilin, Gal Shavit, Kenji Watanabe, Takashi Taniguchi, Yuval Oreg, Tobias Holder, Yuval Ronen

**Affiliations:** † Department of Condensed Matter Physics, 34976 Weizmann Institute of Science, Rehovot 7610001, Israel; ‡ Department of Physics and Institute for Quantum Information and Matter, California Institute of Technology, Pasadena, California 91125, United States; § Research Center for Functional Materials, 52747 National Institute for Materials Science, Tsukuba 305-0044, Japan; ∥ International Center for Materials Nanoarchitectonics, 52747 National Institute for Materials Science, Tsukuba 305-0044, Japan; ⊥ School of Physics and Astronomy, 26745 Tel Aviv University, Tel Aviv 69978, Israel

**Keywords:** twisted trilayer graphene (TTG), moiré heterostructures, orbital magnetism, superconductivity, Josephson junction, valley polarization, time-reversal symmetry breaking

## Abstract

Twisted trilayer graphene (TTG) provides a tunable moiré platform to study correlated phases emerging from flat-band physics. Here, we investigate the interplay between superconductivity and spontaneous orbital magnetism (OM) in alternating TTG devices with intermediate twist angles (1.38–1.44°). Using electrostatically defined Josephson junctions, we demonstrate that OM, stabilized near the charge neutrality point (CNP), competes with gate-induced superconductivity. The OM phase is characterized by sharp jumps in Hall resistance, current-induced bistability, and a Curie–Bloch temperature dependence, indicating broken time-reversal symmetry. Additionally, nonreciprocal Josephson transport—manifested as asymmetric Fraunhofer patterns and a superconducting diode effect—provides independent evidence of an orbital magnetic state confined to the weak link. The observed critical temperature hierarchy, where superconductivity dominates over OM at higher carrier densities and displacement fields, reveals a tunable competition between two broken-symmetry ground states. Our findings establish alternating TTG Josephson devices as a minimal and versatile platform to probe the coexistence of magnetism and superconductivity in engineered moiré systems.

## Graphical abstract

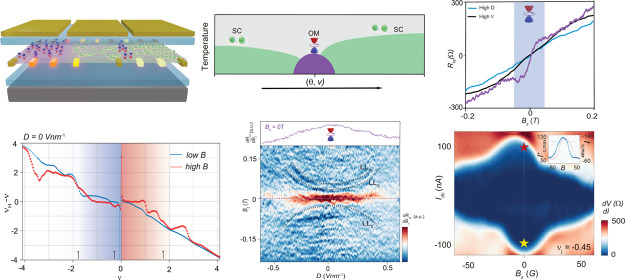

## Introduction

Exploring exotic phases driven by strong electron–electron interactions has led to the discovery of a wide variety of correlated quantum states, including superconductivity, magnetism, and topological order.^1,2^ Flat energy bands—where kinetic energy is quenched—greatly enhance interaction effects, fostering the emergence of such phases.^3–5^ Twisted van der Waals (vdW) heterostructures, which host tunable moiré superlattices, have enabled major breakthroughs in this area, including the observation of superconductivity,^6–8^ orbital magnetism (OM),^3,9–11^ Chern insulators,^12–14^ and more recently, zero-field fractionalized correlated insulators.^15^

Among these phenomena, OM in graphene-based moiré systems has drawn particular interest due to its potential coexistence with superconductivity. Such coexistence is promising for realizing topological superconductivity and non-Abelian excitations in hybrid systems.^16^ However, understanding how superconductivity and OM compete or coexist in moiré platforms remains a significant challenge.

Twisted bilayer graphene (TBG) at the ‘magic angle’ of ∼1.1° was the first system to realize flat bands and correlated insulating and superconducting phases.^6,17,18^ The twist-induced moiré pattern leads to electron localization, suppressing kinetic energy, and amplifying correlations. Twisted trilayer graphene (TTG), with mirror-symmetric top and bottom layers (θ_TM_ = −θ_MB_ = 1.57°), extends this paradigm, producing flat minibands and a Dirac cone inherited from monolayer graphene (MLG).^7,19^ TTG exhibits robust superconductivity over a broad twist angle range (1.2–1.57°), with in-plane critical fields exceeding the Pauli limit,^20,21^ consistent with strong-coupling scenarios.^22^ Intriguingly, superconductivity is suppressed at intermediate twist angles near 1.4°,^21,23^ where the nature of the ground state remains unclear.

OM has also been observed in various twisted graphene heterostructures, including systems with broken inversion symmetry due to hBN alignment,^11,24,25^ spin–orbit proximity from WSe_2,_ ^26,27^ or unaligned off-magic-angle TBG.^28^ However, these platforms typically do not show a stable coexistence of superconductivity and OM, since time-reversal symmetry breaking—required for magnetism—often destabilizes the superconducting phase. Theoretically, superconductivity and OM can emerge in flat-band systems driven by strong interactions. Some recent works have reported OM coexisting with superconductivity in magic-angle TBG^29,30^ and WSe_2_ proximitized TTG.^26^ For instance, Stepanov et al.^29^ reported competing Chern insulating (CI) and superconducting states in TBG, Díez-Mérida et al.^30^ observed diode effects in TBG Josephson junctions (JJs), and Zhang et al.^26^ showed current-driven OM in TTG/WSe_2_ with explicitly broken mirror symmetry. However, the interplay of these phases in mirror-symmetric, unaligned TTG near charge neutrality remains largely unexplored.

In this work, we investigate alternating TTG devices with twist angles of ≈1.38, 1.41, and 1.44°, focusing on the competition between superconductivity and OM. We identify OM via sharp jumps in Hall resistance near zero out-of-plane magnetic field, localized near the charge neutrality point (CNP) on both electron- and hole-doped sides. The phase diagram reveals that the OM is strongest at low densities and displacement fields, while superconductivity dominates at higher densities and large displacement fields. A schematic overview is provided in Figures 1a and 5.

**Figure 1:**
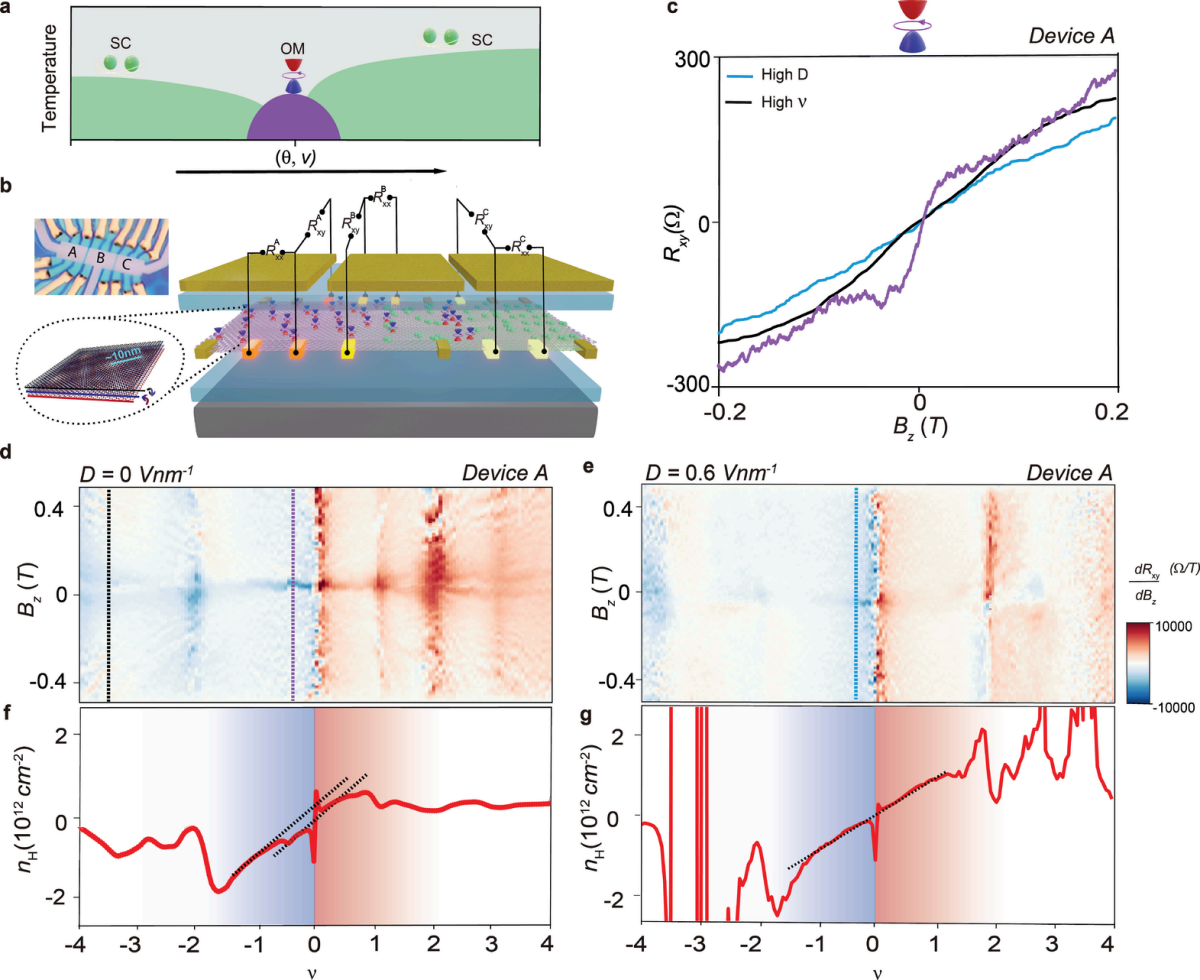
Density tunable jumps in Hall measurements. (a) Schematic phase space of superconductivity and OM in alternating TTG as a function of twist angle θ moiré filling factor *v*. (b) Schematic diagram of alternating TTG Hall bar devices and in situ gate-defined JJs between them. The TTG is encapsulated by hBNs on both sides and metal (Au/Ti) is gated on top and bottom using oxidized silicon (Si/SiO_2_ 285 nm). An enlarged picture of alternating TTG and an optical image of the device are shown alongside. The moiré length is extracted using the formula λ = *a*/[2 sin (θ/2)] (*a* = 0.246 nm and θ = 1.40°). (c) Line scans of *R*_*xy*_ as a function of *B*_*z*_ at three distinct (*v*, D) values, i.e., purple line at ν = −0.45 and *D* = 0 V/nm, black line at ν = −3.6 and *D* = 0 V/nm (high *v*), and blue line at ν = −0.45 and *D* = 0.6 V/nm (high D) for device A. Corresponding colors of line cuts are shown in (d) and (e). (d and e) Landau fan diagram of *B*_*z*_ v/s *v* for differential Hall resistance w.r.t magnetic field $\left(\frac{dR_{xy}}{dB_{z}}\right)$ at *D* = 0 and *D* = 0.6 V/nm, respectively, for device A. (f and g) Variation of Hall carrier density (*n*_H_) with *v* at *B*_*z*_ = 0 at *D* = 0 and *D* = 0.6 V/nm, respectively, for device A.

To probe the TRS-breaking nature of the OM phase, we employ an electrostatically defined JJ, where the weak link is tuned between different correlated regimes. In the OM state, the junction exhibits an asymmetric Fraunhofer interference pattern and a superconducting diode effect—robust signatures of spontaneous TRS breaking localized within the weak link. These features disappear when the weak link is tuned into nonmagnetic or superconducting states, confirming their magnetic origin. The ordering temperature of the OM phase is estimated to be ∼650 mK, notably lower than that of the superconducting transition (∼1.3 K), suggesting a nontrivial competition between the two phases. Our results reveal that alternating TTG at intermediate twist angles hosts a phase diagram distinct from magic-angle TBG, characterized by an unconventional interplay between superconductivity and OM. These findings position TTG as a promising platform for exploring interaction-driven topological phases and designing moiré-based quantum devices with electrically tunable magnetic and superconducting functionalities.

## Results

Our vdW heterostructures consist of alternating TTG, in which the top and bottom graphene layers are twisted mirror-symmetrically relative to the central layer. The TTG stack is encapsulated between hexagonal boron nitride (hBN) layers (25 nm top and 30 nm bottom) and placed atop a Si/SiO_2_ substrate acting as a global back gate. We pattern the heterostructure into three serially connected Hall bars—A, B, and C—using Ti/Au top gates, leveraging the small twist angle variation across the structure to probe OM in different regions (Figure 1b). We also utilized the lithographically defined interfaces between Hall bars A–B (200 nm) and B–C (100 nm) to electrostatically define JJs and study supercurrent flow through the OM regime. Further fabrication details are provided in the Supporting Information (SI), Section 1 and Figure S1.

The carrier density, *n*, and displacement field, *D*, in the TTG are electrostatically tuned by both top and bottom gate voltages (*V*_BG_, *V*_TG_) with the relation determined by their geometric capacitances (*C*_BG_, *C*_TG_), see SI Section 1. The device transport properties were measured in a dilution refrigerator with a base temperature of 10 mK equipped with a 9–1–1 T vector magnet using a standard low-frequency lock-in technique; see methods section and SI Section 1. The twist angles (θ) of the devices are estimated by identifying the moiré superlattice carrier density $n_{s}=8\theta^{2}/\sqrt{3}a^{2}$ (*a* = 0.23 nm), yielding twist angles of 1.38,1.41, and 1.44° for A, B, and C, respectively; see SI Section 1. Based on Landau fan diagram analysis and the position of correlated insulators, the uncertainty is approximately ±0.02°.

The 4-terminal longitudinal (*R*_*xx*_) and transverse (*R*_*xy*_) resistance as a function of carrier density and magnetic field at zero displacement field, *D*, for devices A, B, and C, are shown in SI Figure S2. The *R*_*xx*_ and *R*_*xy*_ were measured using the contact configurations shown in Figure 1b; see SI Section 1. Consistent with prior studies, we observe high-field resistance peaks at integer moiré filling factors (ν = +1, ±2, ±3, ±4) in all devices, indicative of CI states screened by the MLG Dirac spectrum.^8,31^ However, no quantized Hall plateaus are observed at zero magnetic field. Superconductivity is observed in device C and across the JJ between B and C, with a maximum critical temperature *T*_SC_ ∼ 1.3 K (near ν ∼ 2.6 and *D* ∼ 0.38 V/nm); see Figure S8c.

Next, we measured *R*_*xy*_ vs *B*_*z*_ across a wide range of filling factors (ν) and displacement fields. Unexpectedly, at low fields and near the CNP, *R*_*xy*_ exhibits a sharp change in slope (see purple curve in Figure 1c, measured at ν = −0.45 and *D* = 0 V/nm). As either *D* or |ν| increases, the slope gradually returns to a standard linear Hall response. To map the regions where this anomalous Hall slope appears, we plot $\left(\frac{dR_{xy}}{dB_{z}}\right)$ for device A as a function of ν at *D* = 0 and *D* = 0.6 V/nm (Figure 1d,e). Darker regions indicate enhanced slopes near *B*_*z*_ = 0. The signal diminishes with increasing |ν| (Figure 1d) and increasing *D* (Figure 1e). A line cut at ν = −0.45 and *D* = 0.6 V/nm shows this suppression clearly (blue curve, Figure 1c). The resistive peaks at ν = 1, 3, −3 also disappear at high *D*, consistent with suppression of symmetry-broken states. The extracted $\frac{dR_{xy}}{dB_{z}}$ slope, after subtracting the linear Hall background, is shown in the inset of SI Figure S4 for all three Hall bars. In contrast, at high ν (e.g., −3.6), no such enhancement is seen (black line in Figure 1c), consistent with the disappearance of OM signatures beyond |ν| > 2. These findings suggest the emergence of a symmetry-breaking phase near CNP at low displacement fields.

Although we observe no hysteresis in magnetic-field sweeps (Figure S6), OM arising from a time-reversal-symmetry-breaking (TRSB) phase remains a consistent interpretation. Disorder, small symmetry-breaking energy scales, and competing interactions can smear putative first-order transitions and obscure hysteresis. Similar behaviors have been reported in few-layer WS_2_ under pressure^32^ and KV_3_Sb_5_,^33^ where anomalous Hall effects appear without measurable hysteresis. Furthermore, screening from coexisting Dirac-like dispersive bands can reduce the strength of interactions, diminishing magnetic response.^26,34^

To further examine the electronic reconstruction, we extracted the Hall carrier density $n_{H}=1/e\times \frac{dR_{xy}}{dB_{z}}$ at *B*_*z*_ = 0 (see SI Section 2 and Figure S3). Figure 1f shows *n*_H_ vs *v* plot at *D* = 0 V/nm, where we observe resets at integer ν = 1, ±2, ±3, ±4, consistent with correlated insulating states stabilized by flavor polarization.^19,35^ However, instead of a linear slope in *n*_H_ vs *v*, an offset is observed from both doping regions of the CNP, emanating from fractional fillings of ν ∼ −0.5 and 0.7. These deviations suggest an underlying reconstruction of the density of states (DOS), potentially arising from a spontaneous symmetry breaking of degenerate bands near CNP that produces an intrinsic effective magnetic field.^36,37^ When the same analysis is repeated at higher displacement field (*D* = 0.6 V/nm), as shown in Figure 1g, the Hall carrier density follows the expected linear dependence around the CNP, indicating a suppression of the spontaneous symmetry-breaking state at high *D*.

The mirror-symmetric configuration of graphene layers in TTG enables the exploration of hybridization effects between the Dirac and flat-band sectors by using a displacement field. Figure 2a,b shows the phase space of $\frac{dR_{xy}}{dB_{z}}$ as a function of *B*_*z*_ and *D*, at ν = −0.45 and 0.7, respectively, for device A. Darker regions represent larger magnitudes of $\left|\frac{dR_{xy}}{dB_{z}}\right|$ . In Figure 2a, at ν = −0.45, a jump in *R*_*xy*_ is observed at *D* = 0 V/nm (indicated by the brown color), as also seen in Figure 1c. This jump is progressively suppressed as the displacement field increases beyond |*D*| > 0.5 V/nm. In Figure 2b, at ν = 0.7, an opposite-sign slope in *R*_*xy*_ is observed, with jumps in *R*_*xy*_ (indicated by the blue color) being suppressed for |*D*| > 0.25 V/nm. These findings suggest that increasing *D* induces hybridization between the monolayer-like Dirac sector and the flat bands, thereby destabilizing the orbital magnetic phase.

**Figure 2:**
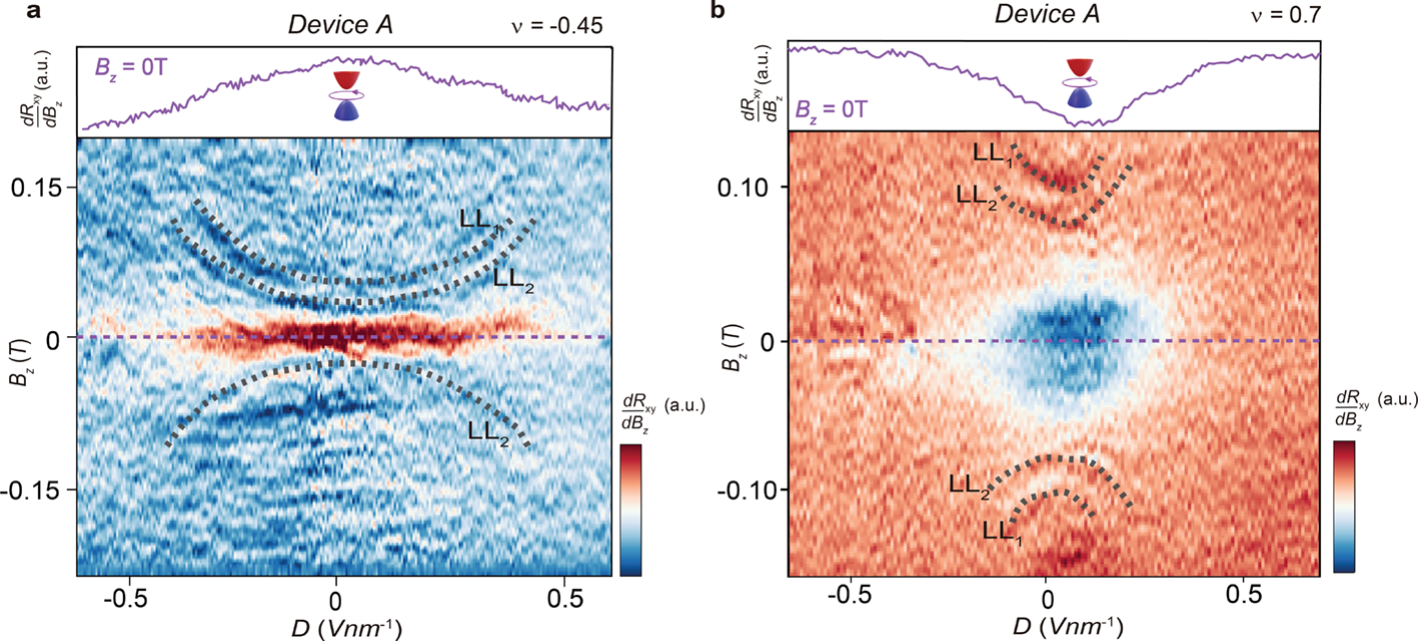
Displacement field tunable jumps in Hall measurements. The displacement field (*D*) dependence of $\frac{dR_{xy}}{dB_{z}}$ while sweeping *B*_*z*_ around 0T for device A. The moiré filling factor (*v*) is fixed at (a) *v* = −0.45 and (b) *v* = 0.70. The dark brown (a) and blue regions (b) nearby *B*_*z*_ = 0 T show the maximum change in $\frac{dR_{xy}}{dB_{z}}$ , which disappear at high *D*. Insets show the line cuts at *B*_*z*_ = 0 T, corresponding to purple dash lines in figures. The dotted, curved black lines represent the variation of Landau levels 1 (LL_1_) and 2 (LL_2_) of the monolayer Dirac sector with D.

Band mixing is thought to play a crucial role in determining the nature of the correlated low-energy phases.^38,39^ To quantify the hybridization of the two sectors, we analyzed the parabolic dashed curves highlighted in Figure 2. These curves correspond to the Shubnikov–de Haas (SdH) oscillations of the MLG Landau levels (LL_s_), at specific Fermi energies *E*_F_ for a constant *v*.^6,31,39^ These MLG LL_s_ provide a spectroscopic probe of the Dirac dispersion, and by examining the shift of them, we can infer the degree of hybridization between the two sectors. If we assume the Fermi velocity, *v*_F_ = d*E*/d*k* of MLG Dirac cone is 10^6^ m/s at *D* = 0 V/nm, we can estimate the percentage change in *v*_F_ with increase in *D*, see SI Section 2 and Figure S5 for more details. At ν = −0.1, −0.45, and −0.70, for an increase in *D* from 0 to 0.20 V/nm, *v*_F_ decreases by ∼30, 20, and 12% respectively. The MLG LL curvature lines disappear at *D* ∼ 0.45, 0.40, and 0.25 V/nm at =–0.1, −0.45, and −0.70 respectively, coinciding with the disappearance of OM, see Figures 2a,b, and S5a. These observations suggest that near the CNP, a higher *D* is required to hybridize the MLG Dirac cone with the flat bands, allowing OM to persist over a wider range of displacement fields. In contrast, at higher carrier densities, smaller values of *D* are sufficient to induce hybridization, as the Fermi level approaches the energy overlap between MLG and flat bands.^31^ Interestingly, ref 38 predicts the emergence of singlet superconductivity near CNP under low displacement fields in TTG. The observation of OM near CNP in our samples may explain the absence of such a superconducting state, pointing to competing symmetry-breaking orders in this regime.

Magnetic ordering arises from exchange interactions that break TRS and can originate from either spin magnetic moments or orbital magnetic moments driven by electron circular motion. While spin magnetism is typically isotropic, OM is known to exhibit a strong anisotropy. Previous studies have indicated that magnetism in graphene-based heterostructures is predominantly of orbital origin.^11,23,24,28,30^ Although antiferromagnetic (AFM) ordering has been theoretically proposed in TTG near charge neutrality,^40^ such states are generally spin-based and respond weakly to in-plane magnetic fields, in contrast to OM, which is highly sensitive to the orientation of the applied field.^24^ To distinguish between these possibilities, we performed angle-dependent Hall measurements, as described below.

Figure 3a shows *R*_*xy*_ as a function of *B*→ = *B* cos­(θ)·*ẑ* + *B* sin­(θ)·*x̂* at *v* = −0.45 and *D* = 0 V/nm for device A. The angle θ between the sample and *B*→ is varied from 90 to 0° in the steps of 15°. The steepest slope in *R*_*xy*_ is observed when *B*→ = *B*_*z*_·*ẑ*, while no jump is detected when *B*→ = *B*_*x*_·*x̂*, indicating the highly anisotropic nature of the magnetic state. See Figure S7 of the SI for the same measurements on devices C and D. Supplementary Figure S4a,b shows *R*_*xy*_(*B*_*z*_) on the electron- and hole-doped sides of charge neutrality at *D* = 0 V/nm. The jump amplitude is maximal near ν ≈ −0.45 and shows a secondary maximum near +0.70; it then decreases steadily with |ν|—already small by |ν| ≈ 1 and indistinguishable from background by |ν| ≈ 2. The inset quantifies this by plotting |d*R*_*xy*_/d*B*_*z*_| near *B*_*z*_ = 0 versus ν for all three devices (see SI Figures S4 and S5 for more discussion). The inset of Figure 3a shows *R*_*xy*_ measured as a function of *B*_*z*_ while a fixed *B*_*x*_ = 0, 0.5, and 1 T is applied. The amplitude of the *R*_*xy*_ jump remains unchanged even with *B*_*x*_ applied up to 1 T. This pronounced out-of-plane anisotropy—combined with the insensitivity to in-plane magnetic fields—provides compelling evidence that the magnetic state is orbital in origin.

**Figure 3:**
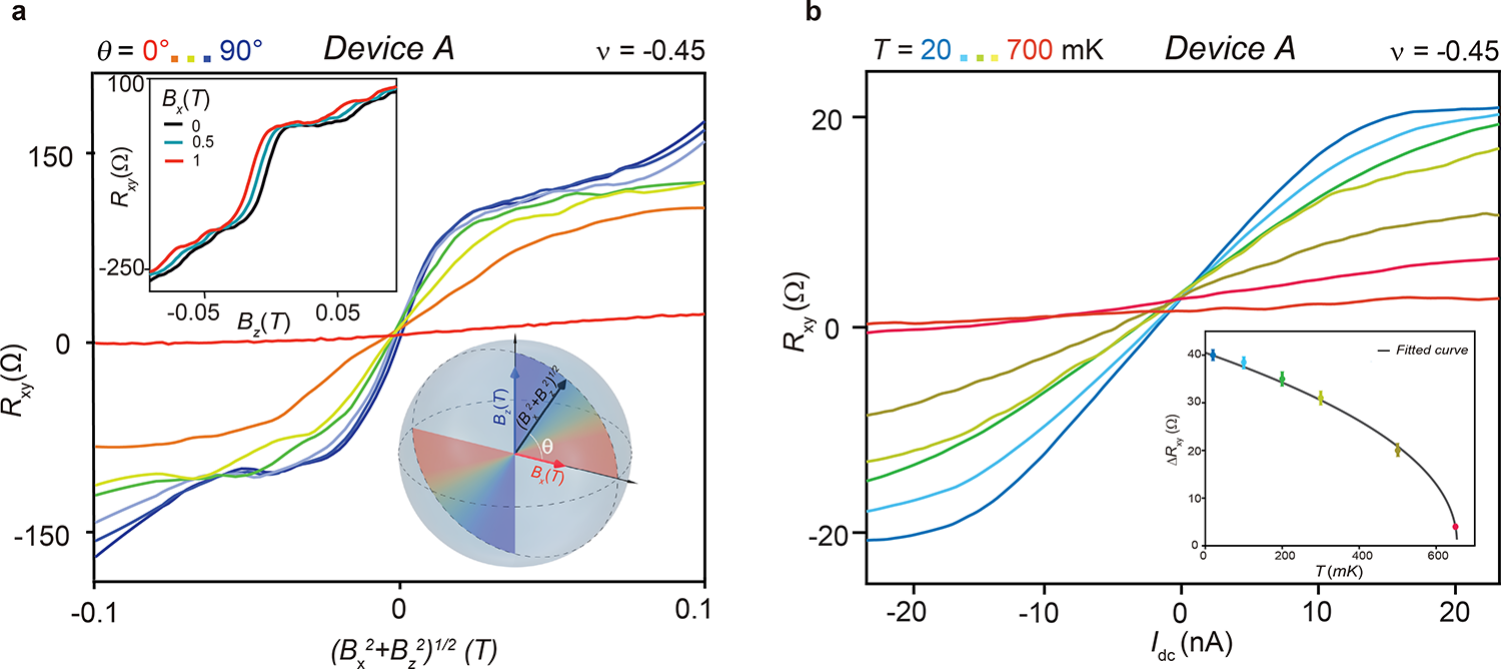
Confirming the orbital nature of the jumps in *R*_*xy*_. (a) *R*_*xy*_ vs *B* = (*B*_*x*_^2^ + *B*_*z*_^2^)^1/2^ at varying θ between sample and *B* from 90 (blue) to 0°(red) in steps of 15° for device A. Inset shows the jumps in *R*_*xy*_ as a function of *B*_*z*_ at constant in-plane field *B*_*x*_ = 0, 0.5, and 1 T. (b) Temperature dependence of jumps in *R*_*xy*_ as a function of DC bias current (*I*_dc_) at temperatures ranging from 20 (blue) to 700 mK (red) measured at *B*_*z*_ = 0 T for device A. Inset shows a fit of the jump height in *R*_*xy*_ (Δ*R*_*xy*_) to the Curie Bloch equation: Δ*R*_*xy*_ = *p*(1 – *T*/*T*_OM1_)^α^.

In studies on TBG aligned with hBN, orbital magnetic moments were polarized using an external DC bias (*I*_dc_) superimposed on a small AC signal.^11,25^ A similar approach was recently extended to TTG coupled to few-layer WSe_2_, where DC current-induced switching of *R*_*xy*_ was observed and attributed to OM driven by TRS breaking.^26^ Inspired by these findings, we measured *R*_*xy*_ at *v* = −0.45 and *D* = 0 V/nm with an AC excitation of approximately 1 nA while varying the *I*_dc_ from −25 to +25 nA at *B*_*z*_ = 0 T. A jump in *R*_*xy*_ is observed at 20 mK, and the amplitude of this jump decreases with increasing temperature. Figure 3b shows the measurements taken up to 700 mK, with no jump observed above approximately 650 mK. The difference in *R*_*xy*_ at *I*_dc_ = −20 and +20 nA, i.e., Δ*R*_*xy*_ can be fitted to the Curie Bloch equation Δ*R*_*xy*_ = *p*(1 – *T*/*T*_OM1_)^α^ (here, *p* is a proportionality constant, α is the fitting exponent, and *T*_OM1_ is the critical temperature of the magnetic state) yielding *T*_OM1_ = 654 ± 6 mK, *p* = 40.5 ± 1 Ω, and α = 0.45 ± 0.05.

These bias-induced jumps in Hall resistance indicate the coupling between electric fields generated by the transport current and the underlying magnetization of sample.^41^ Similar switching observed in TBG/hBN systems has been linked to the interplay of extrinsic sublattice symmetry breaking (from hBN alignment), rotational symmetry breaking (e.g., from strain), and intrinsic TRS breaking.^41^ In TTG, comparable mechanisms are likely at play. Recent experiments have demonstrated broken 3-fold rotational symmetry (*C*_3_) in TTG via nonreciprocal transport^26^ and have identified a nematic semimetallic ground state near charge neutrality.^31^ Our results implicate the orbital magnetic moments, due to the spontaneous breaking of valley isospin symmetry near the CNP, as the culprits in facilitating the significant current-magnetization coupling. Furthermore, this interpretation aligns with recent theoretical and experimental findings that highlight the role of exchange interactions in stabilizing correlated magnetic states for *v* < 1.5, whereas at higher densities, the dominant energy scale shifts to charging self-energy effects.^31^ The robustness of the bias-induced switching and its sensitivity to temperature and carrier density provide strong evidence for current-magnetization coupling driven by orbital magnetic moments in TTG.

We now investigate the impact of OM on supercurrent flow by incorporating it as the weak link in an electrostatically defined JJ,^42,43^ leveraging the tunable superconductivity in TTG. In previous twisted graphene devices, valley-polarization-driven OM has been shown to generate asymmetric Fraunhofer patterns.^21,30^ SI Figure S9a–c presents the *R*_*xx*_ measurements for all three devices under varying displacement fields. As reported earlier, superconductivity is enhanced at high *D* in device C (1.44°, see SI Figure S9c)^7,8^ while suppressed in devices with twist angles between 1.38 and 1.41°, consistent with earlier studies.^21,23^ Additionally, we confirm that a section of device B also becomes superconducting under appropriate gate conditions: line cut of the transverse resistance *R*_*xy*_ in device B (contacts 12–5, *D* = 0.6 V/nm, and *T* = 20 mK) shows a clear zero-resistance plateau between ±2 < ν < ±3 (see SI Figure S8d). This provides direct evidence that the region of device B participating in the JJ is indeed superconducting under the operating gate conditions. To probe the OM-supercurrent interplay, we created a JJ using the interface between devices B and C (JJ2, contacts 5–6; see Supplementary Figure S1). The phase space of *R*_*xx*_ for this junction is shown in SI Figure S10a. Clear resistive and superconducting states are observed across the junction, indicating a percolating superconducting path. In the (*V*_TG_, *V*_BG_) map (SI Figure S10a), a slanted superconducting stripe appears when both leads lie inside the |ν | ≈ 2–3 dome while *V*_BG_ tunes the link; if either lead were not superconducting, this stripe would terminate. Outside this stripe, the device remains resistive (finite *R*_*xx*_), consistent with the absence of a continuous superconducting path when either lead leaves the dome. The yellow dashed lines represent the left and right sides of JJ, gated using both top and back gates, while the vertical pink lines correspond to the weak link region (filling factor *v*_*j*_), gated solely by the back gate. By sweeping both gates independently, we can electrostatically define the superconducting leads and the weak link, confirming that superconductivity arises from gate-defined regions within each device. This gating configuration also enables us to explore the phase boundaries between superconductivity and OM across the junction.

Figure 4a–c shows the critical current as a function of the out-of-plane magnetic field, *B*_*z*_, for different phases of the JJ weak link. The differential resistance (d*V*/d*I*) was measured at 20 mK with an AC excitation of 1 nA while sweeping the DC component of the current, *I*_dc_. The left and right sides of JJ are tuned to a superconducting state (S) corresponding to *v* ∼ 2.6 by using a combination of top and bottom gates. The weak link is tuned to a superconducting state (*S*′) *v* *_j_* ∼ −2.7 (*D*_*j*_ ∼ −0.3 V/nm), normal metallic state (N) *v*_j_ ∼ −1.7 (*D*_*j*_ ∼ −0.19 V/nm), and orbital magnetic state (OM) *v*_j_ ∼ −0.45 (*D*_*j*_ ∼ −0.05 V/nm) using the back gate only, as shown in the insets of Figure 4a–c, respectively. The line cuts of d*V*/d*I* vs *I*_dc_ at *B*_*z*_ = 0 G for these JJ configurations are shown in SI Figure S8a.

**Figure 4:**
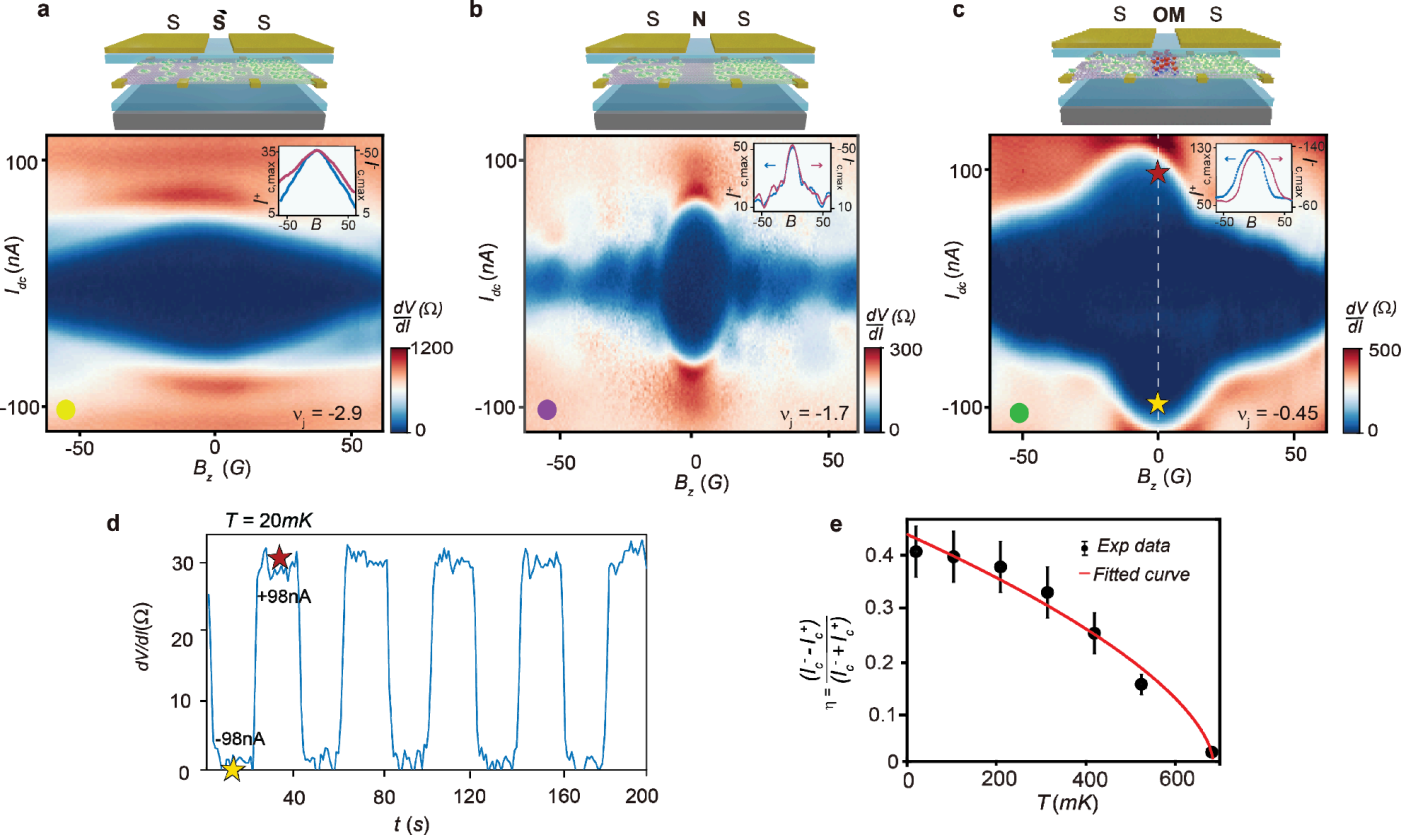
Gate defined the JJ as a probe for OM. The Fraunhofer pattern measurements across JJ2 (contacts 5–6, see SI Figure S1) and left and right sides of JJ tuned to superconducting state (S) at *v* ∼ 2.6 and weak link tuned to (a) *v*_j_ ∼ −2.7 forming S|S^′^|S JJ, (b) *v*_j_ ∼ −1.7 forming S|N|S JJ, and (c) *v*_j_ ∼ −0.45 forming S|OM|S JJ. Insets on top of the figures show the schematics corresponding to JJ configurations. Top right insets show the line cuts of the positive SC critical current (*I*_c,max_^+^) and negative SC critical current (*I*_c,max_^–^) extracted from corresponding Fraunhofer patterns. (d) d*V*/d*I* (Ω) characteristics of S|OM|S JJ measured at *B*_*z*_ = 0 G while alternating *I*_dc_ between *I*_c_^–^ (−98 nA) and −*I*_c_^–^ (+98 nA) after every 20 s at 20 mK. (e) Measure of asymmetry in critical currents $\eta =\frac{\left(I_{C}^{-}-I_{C}^{+}\right)}{\left(I_{C}^{-}+I_{C}^{+}\right)}$ is plotted as a function of temperature (black data points) at *B*_*z*_ = 0 G. The red line corresponds to fit the Curie Bloch equation η = *k*(1 – *T*/*T*_OM2_)^β^.

Figure 4a presents a typical 2D map for the S|S′|S configuration, where the absence of a Fraunhofer pattern confirms a spatially uniform superconducting phase across the junction. As expected in this configuration, the maximum positive superconductivity critical current (*I*_c,max_^+^) and maximum negative superconductivity critical current (*I*_c,max_^–^) curves are symmetric along *B*_*z*_ axis, see inset of Figure 4a. In contrast, the S|N|S configuration (Figure 4b) exhibits a clear Fraunhofer interference pattern, consistent with a nonsuperconducting weak link. In narrow thin-film (2D) JJs, the flux periodicity of Fraunhofer oscillations differs significantly from that in 3D junctions of comparable width. For 2D superconductors based JJ, the high-field periodicity of Fraunhofer oscillations is given by $\Delta B\approx \frac{\pi \phi_{0}}{4a_{0}w^{2}}$ ; where ϕ_0_ is the magnetic flux quantum, *w* is the junction width, and *a*_0_ is a geometric factor derived from fitting the interference pattern using Bessel function modeling.^44^ As shown in SI Figure 10d (cyan line), we fit experimental oscillations using this equation with *a*_0_ ≈ 0.5, obtaining a flux periodicity Δ*B* ≈ 10 G and an effective width *w* ≈ 1.9 μm, which closely matches the lithographically defined junction width of ∼2 μm. The central lobe exhibits a broader width of ∼15 G, slightly larger than the extracted high-field periodicity. This deviation may reflect geometric effects such as partial flux focusing or nonuniform field distribution in the junction region.^42–45^ Additionally, the amplitude decay of the critical current lobes follows the expected $\propto 1/\sqrt{B}$ trend (white line in SI Figure 10d), as predicted for ballistic JJs in 2D superconductors, further validating the coherence and uniformity of the weak link. Similar deviations from ideal periodicity have been observed in gate-defined JJs in TBG,^42,43^ further validating the Josephson nature of our device. From the S|N|S period Δ*B* ≈ 10 G, we obtain *w*_eff_ ≈ 1.9 μm via the 2D-JJ expression, matching the ∼2 μm lithographic channel; a broad fringe-field spillover would enlarge *w*_eff_ and substantially reduce Δ*B*, which we do not observe. As an off-junction control, a device-C Hall-bar segment (measured at the S|N|S gate setting) shows a smooth, nodeless *I*_c_(*B*_*z*_), confirming that the Hall bar does not host an unintended weak link, see Figure S12c.

In stark contrast, the S|OM|S configuration (Figure 4c) exhibits a pronounced asymmetry in the Fraunhofer pattern: *I*_c,max_^+^ ≠ *I*_c and max_^–^ as a function of *B*_*z*_ axis. This asymmetry decreases with increasing temperature and vanishes around 650 mK (SI Figure S10c), consistent with the Curie temperature extracted from DC current switching. Although the minima are shallow, estimating the spacing between successive minima/inflection points yields a modulation period of ∼15 G (*w*_eff_ ≈ 1.6 μm)—comparable to S|N|S—indicating that the active width is not enlarged by a broad interfacial gradient. Additional confirmation comes from SI Figure S10e,f. SI Figure 10e shows asymmetric current–voltage (*I–V*) characteristics across the full junction in the S|OM|S configuration at *B*_*z*_ = 0. We also observe a smaller but discernible asymmetry in the normal-state resistance (*R*_N_) which we interpret as arising from valley-polarized scattering in the orbital magnetic phase near charge neutrality (this asymmetry is not forbidden by Onsager reciprocity, which allows σ*_*xx*_* (B, M) ≠ σ*_*xx*_* (−B, M) when TRS is broken). The OM state breaks TRS and induces direction-dependent transport, particularly at low densities where interaction-driven anisotropies in the Fermi surface (e.g., nematicity or valley imbalance) may emerge.^26,31^ This observation is further supported by the symmetric critical current and normal resistance in the S|*S*′|S and S|N|S configurations (Figure 4a,b), where the weak link lies outside the OM regime, suggesting that the asymmetry is linked to the orbital magnetic state rather than device geometry. While the asymmetry in *R*_N_ is consistent with broken TRS, we do not interpret it as conclusive evidence for OM, as such nonreciprocal responses could also arise from extrinsic scattering. SI Figure 10f shows that the right superconducting region (device C), measured independently, remains symmetric, confirming that the superconducting leads themselves do not break TRS.

To further verify spontaneous TRS breaking, we performed superconducting diode effect measurements at *B*_*z*_ = 0. We cyclically drove the junction from the superconducting to the normal state by alternating *I*_dc_ between ±98 nA (white dashed line, Figure 4c). The resulting differential resistance d*V*/d*I*, measured at 20 mK and plotted over time in Figure 4d, exhibits a clear nonreciprocal response: the junction switches between 0 Ω (superconducting) and 29 ± 4 Ω (normal state), consistent with a superconducting diode effect. This behavior disappears at 650 mK (see SI Figure S11a), reinforcing the temperature scale of the underlying TRS-breaking state. In Figure 4e, we quantify the asymmetry of the Fraunhofer pattern using the diode efficiency parameter $\eta =\frac{\left(I_{C}^{-}-I_{C}^{+}\right)}{\left(I_{C}^{-}+I_{C}^{+}\right)}$ , where *I*_C_^–^ and *I*_C_^+^ are the critical currents measured across for various temperatures (see SI Figure S8b). As the temperature increases, η gradually decreases, reaching zero at 650 mK, where the Fraunhofer pattern becomes symmetric, see SI Figures S10b,c for additional details. By fitting η to the Curie Bloch equation, η = *k*(1 – *T*/*T*_OM2_)^β^, where *k* is a proportionality constant, *T* is the temperature, and *T*_OM2_ the critical temperature, we obtained the following parameters: *k* = 0.44 ± 0.06, β ∼ 0.6 ± 0.2, and *T*_OM2_ ∼ 650 ± 20 mK. These results are in good agreement with the TRS-breaking behavior observed via diode effect switching and the *R*_*xy*_ vs *I*_dc_ fits in Figure 3b, further supporting the connection to OM. Crucially, both the diode effect (Figure 4d) and the asymmetric *I*–*V* characteristics were measured at *B*_*z*_ = 0, directly confirming that the nonreciprocity originates from a spontaneously broken time-reversal symmetric ground state, independent of any external magnetic field.

This strongly suggests that the observed asymmetry in the Fraunhofer pattern in the S|OM|S configuration arises from an orbital magnetic state, possibly involving magnetic domain boundary motion within the weak link. Importantly, control measurements on other configurations (e.g., S|S′|S and S|N|S; Figure 4a,b) show symmetric critical currents, ruling out device-intrinsic effects as the source of the nonreciprocity.^30,46^

## Discussion

We observed OM in TTG devices of intermediate twist angles of 1.38 and 1.41°, in the vicinity of CNP with no clear signs of superconductivity in these samples.^21,23^ In contrast, a third device with a slightly larger twist angle of 1.44°—closer to the magic angle—exhibits both superconductivity and OM. Notably, transport measurements across the contact pair used to define the JJ confirm that the part of device B participating in the JJ is superconducting under the applied gate voltages. To probe the orbital magnetic state, we employed two independent techniques: (1) low-field Hall effect measurements and (2) JJ transport. Both probes revealed consistent signatures of OM, with switching behavior that follows a Curie–Bloch temperature dependence and yields a common magnetic ordering temperature *T*_OM_ ≈ 650 mK. Notably, our devices exhibit an inverted energy hierarchy compared to prior reports, with OM emerging at a lower onset temperature than superconductivity (*T*_SC_ ∼ 1.3 K). The JJ is dissipative at low temperatures, and upon heating above *T*_OM_, resistance drops by orders of magnitude, allowing superconductivity to prevail. This reversal in energy scales is unusual and suggests that the spontaneous valley-polarized OM in TTG may arise from a different mechanism than the anomalous Hall effect observed at integer fillings in other twisted graphene systems.^11,25,26,28,29^ To sharpen phase identification and isolate the weak link in future experiments, we will implement an independently addressable finger top gate over the junction, using high-quality ALD Al_2_O_3_ (HfO_2_ also compatible), enabling local control of (*n*, *D*) in the link without perturbing the superconducting leads and minimizing fringe-field spillover.

We systematically mapped the phase space of OM as a function of filling factor *v* and displacement field *D*. As shown in Figure 5a, OM is most robust near the CNP and fades as *v* → ±2, where superconductivity emerges. Increasing the *D* concentration weakens the OM while enhancing the superconductivity, indicating a complementary relationship between the two phases. Using MLG LLs as probes, we further demonstrated that this crossover arises from hybridization between Dirac and flat-band sectors, with higher displacement fields enhancing hybridization and suppressing OM.^31,38,40^ This hybridization is directly visible in the suppression of Dirac LL curvature and reduction in Fermi velocity (Figure 2), consistent with theoretical expectations.^31^

These ordering tendencies align with the band structure of TTG: at the magic angle and zero displacement field, the Dirac cone remains decoupled from the flat moiré mini bands. Detuning from either condition increases the bandwidth of the flat bands, raising the Fermi velocity and lowering the DOS. In such regimes, interaction-driven flavor polarization (i.e., isospin symmetry breaking) is energetically favored, while more delicate intervalley coherent (IVC) states—such as Kekulé spiral orders—typically stabilize only in ultraflat bands near the magic angle. Thus, the emergence of OM at intermediate twist angles reflects a general trend toward isospin-polarized states in bands that are not perfectly flat.^28,47–49^ This is supported by prior theoretical studies in TBG showing that valley polarization as a common ground state at low fillings under realistic conditions, including strain and interactions.^50,51^ The associated spontaneous breaking of TRS provides a natural origin for the OM observed in our samples.

Our phase diagram is further supported by a comparison of twist-angle-dependent data from our experiments, and the literature, see Figure 5b. Around twist angles of ∼1.40°, OM spans a broad density range while superconductivity is absent. In contrast, devices further from this regime favor superconductivity over OM. To validate this trend, we measured two additional TTG devices with twist angles of ∼1.3 and 1.5°, both of which showed clear superconductivity but no OM (see SI Section 3 and Figures S13–S15). The absence of OM in the 1.50° device aligns with prior results in near-magic-angle TTG, while the 1.3° device may fall outside the interaction-to-bandwidth (U/W) regime required to stabilize OM.

**Figure 5:**
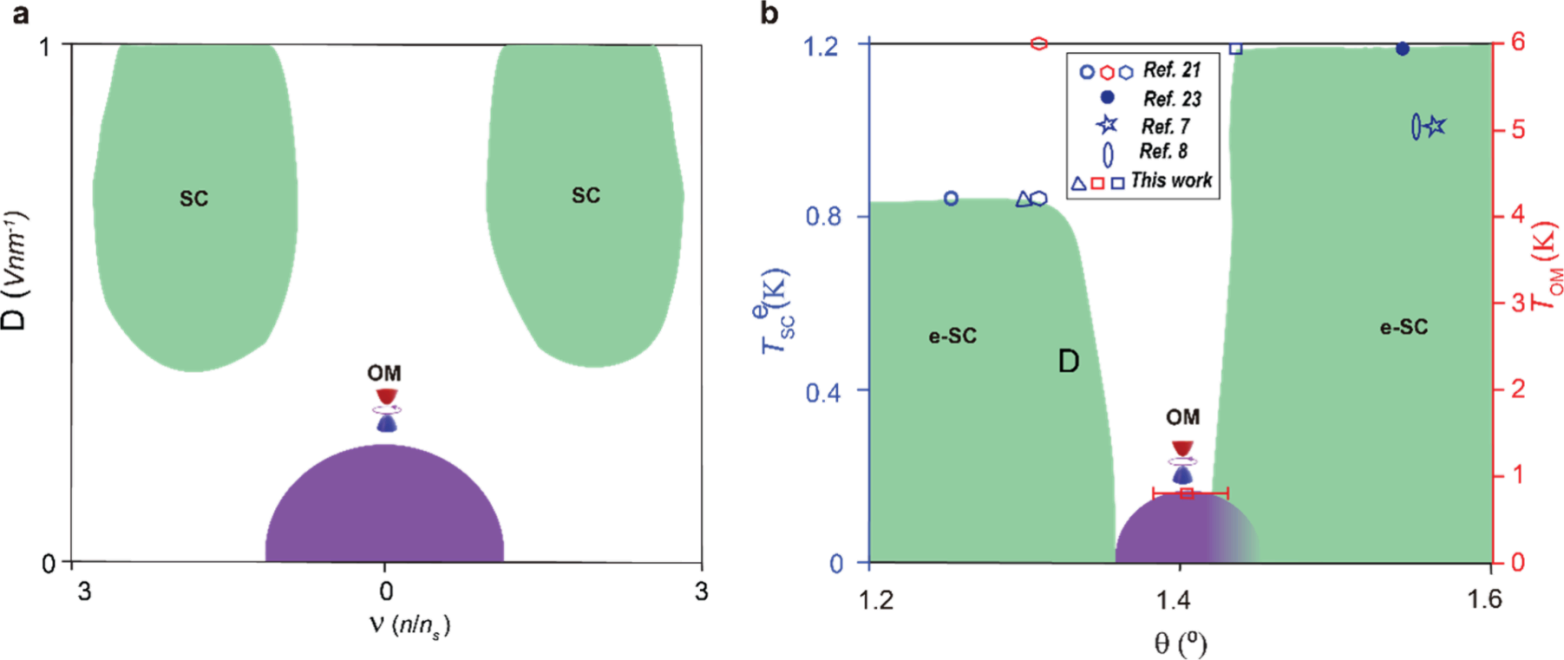
Phase diagrams of superconductivity and OM as a function of twist angle, density, and displacement field. (a) General phase diagram of SC and OM with *v* and displacement field *D*. The OM is stronger near *D*(*v*) = 0, and gets weaker as we move away, whereas SC is weaker (absent) near *D*(*v*) = 0 and gets stronger as we move away. (b) Phase space of critical temperature of electron-doped superconductivity (e-SC) *T*_SC_^e^ (blue data points, corresponding to left *y* axis) and OM ordering temperature *T*_OM_ (red data points corresponding to right *Y* axis) as a function of twist angle θ for alternating TTG devices. The OM peaks around 1.40° and is flanked by e-SC domes on lower and higher angles.

The spontaneous nature of TRS breaking in the OM regime is supported by three key observations: (1) discrete jumps in *R*_*xy*_ vs *B*_*z*_ near the CNP; (2) current-induced switching of *R*_*xy*_ at *B*_*z*_ = 0 (Figure 3b); and (3) diode effect in the S|OM|S JJ, manifesting as nonreciprocal transport *B*_*z*_ = 0 (Figure 4d). These effects disappear above 650 mK (Figure 4e), confirming *T*_OM_ as a consistent transition scale. The lack of magnetic hysteresis in *R*_*xy*_ does not contradict OM; it may reflect disorder, weak energy scales, or screening effects that soften first-order transitions. This interpretation is consistent with reports of OM without hysteresis in TTG/WSe_2_ and layered systems like WS_2_.^26,32,33,37^

Although AFM ordering has been proposed near CNP in TTG,^40^ such spin-based orders are typically isotropic and cannot explain the pronounced field-direction dependence seen in *R*_*xy*_. Our angle-dependent Hall data (Figure 3a) reveal highly anisotropic behavior, favoring an orbital, not spin-based, magnetic origin. Moreover, OM persists even at *D* = 0, where mirror symmetry is preserved, implying that spontaneous valley polarization is sufficient to stabilize the magnetic state. Still, we cannot rule out contributions from residual strain, which may break rotational symmetry and influence the ground state.^26,40,52,53^

We also considered a two-carrier model involving coexisting electron and hole pockets near the CNP. Such models predict smooth, convex Hall curves with continuous slope variation,^54^ unlike our sharp, discrete Hall slope jumps near ν = 0 (Figures 1c and S4). Moreover, TTG strongly breaks particle–hole symmetry,^55^ making electron–hole compensation unlikely. The diode effect and asymmetric Fraunhofer pattern—both at zero field—further support a spontaneously TRS-broken OM phase and disfavor two-carrier or trivial transport explanations.

Our findings extend previous studies of OM and superconductivity in moiré graphene. While Stepanov et al.^29^ reported CI and superconducting states in magic-angle TBG, Díez-Mérida et al.^30^ showed diode effects in symmetry-broken TBG junctions, and Zhang et al.^26^ demonstrated OM in WSe_2_ aligned TTG with broken mirror symmetry, our work shows spontaneous OM at fractional fillings in mirror-symmetric, unaligned TTG. This highlights the generality of interaction-driven OM, independent of spin–orbit proximity or alignment, and establishes alternating TTG at intermediate twist angles (1.38–1.44°) as a tunable platform to explore the interplay between OM and superconductivity.

## Conclusions

Our measurements uncover a distinct phase diagram in alternating TTG, where superconductivity and OM compete as functions of twist angle, carrier density, and displacement field. Unlike magic-angle TBG^29^ and TTG/WSe_2_ ^26^ heterostructures, which typically host superconductivity at |ν| ≈ 2–3 and OM at integer fillings with higher onset temperatures, our unaligned TTG devices exhibit OM near charge neutrality at fractional fillings, with a lower onset temperature than superconductivity.

This reversed energy hierarchy—where OM precedes superconductivity—points to a nontrivial ground state, potentially involving valley ferromagnetism or critical magnetic fluctuations influencing pairing. The tunable hybridization between Dirac and flat bands via a displacement field plays a key role in stabilizing these phases, with OM and superconductivity occupying complementary regions of the phase diagram.

More broadly, our results position intermediate-angle TTG as a minimal and tunable platform for investigating the coexistence and competition of correlated electronic phases. This opens pathways for realizing topological superconductivity and exploring non-Abelian quasiparticles in a vdW-based Josephson architecture. As an immediate outlook, angle control provides a clean knob to traverse OM- and SC-dominated regimes; in future devices, we will implement an independently biasable finger top gate (ALD Al_2_O_3_/HfO_2_) over the weak link to map the link-only (*n*, *D*) phase space and to test the evolution of nonreciprocity across the OM–SC boundary.

## Methods

### Stacking and Device Fabrication

The TTG stack is prepared using the dry-transfer method. The hBN and graphene flakes are exfoliated on clean Si/SiO_2_ (285 nm) substrate. The number of graphene layers are determined by examining the fwhm of 2D peak Raman spectra (WITec alpha300R) using a 532 nm laser. MLG ∼100 × 30 μm is cut into three pieces separated by ∼5 μm gap using 1064 nm Raman laser. The clean hBN crystals are examined using an optical microscope and dark field microscopy. The crystallographic axes of hBNs are determined by using straight edges. Stamps for picking the flakes are prepared by placing polycarbonate (PC) thin films on polydimethylsiloxane (PDMS) dome stamps. The top hBN flake (∼25 nm) is picked at 100 °C, and the graphene flakes are picked up at 40 °C. The transfer stage holding the Si chip with vacuum is rotated to ∼1.45 and ∼−1.45° to obtain the mirror-symmetric configuration of TTG. The bottom hBN (∼30 nm) is picked up at 50 °C and the final stack dropped on clean Si/SiO_2_ (285 nm) substrate at 180 °C. The melted PC on the stack is cleaned using chloroform and stack annealed in a vacuum at 350 °C to move the air bubbles, release strain, and remove impurities on top of the stack. The stack’s contact mode cleaning and hBNs’ thicknesses are determined using a Bruker atomic force microscope. Jeol JBX9300-FS e-beam lithography is used to define metal top gates and JJs of lateral width ∼2 μm. The Cr­(4 nm)/Au­(16 nm) metals are deposited with an e-gun evaporator for top gates. The edge contacts to graphene are made using CHF_3_/O_2_ plasma in RIE and consecutively depositing the Cr­(2 nm)/Au­(65 nm) metals in the angle rotator e-gun evaporator. The device is etched into Hall bar geometry using CHF_3_/O_2_ plasma in the RIE.

### Measurements

Bluefors LD400 dilution refrigerator with RC and RF filtering having base temperature ∼10 mK is used to measure the transport characteristics. The Q-devil sample puck with additional filtering is used to mount the sample to the fridge. The four-probe measurements are performed using standard lock-in techniques using *I*_ac_ ∼ 1 nA rms (100 MΩ resistor) and 11.377 Hz frequency. The Femto voltage amplifiers are used at room temperature to amplify signals from fridge to SRS830 and SRS865A lock-in amplifiers. Keithley 2400 source meters are used to apply top and bottom gate voltages. The bottom gate voltage (*V*_bg_) and top gate voltage (*V*_tg_) are converted to (*n*) and (*D*) using electrostatic equations $n=\frac{\varepsilon_{b}\varepsilon_{0}(V_{\mathrm{bg}}-V_{\mathrm{bo}})}{ed_{b}}+\frac{\varepsilon_{t}\varepsilon_{0}(V_{\mathrm{tg}}-V_{\mathrm{to}})}{ed_{t}}$ and $D=\frac{\varepsilon_{b}\varepsilon_{0}(V_{\mathrm{bg}}-V_{\mathrm{bo}})}{d_{b}}-\frac{\varepsilon_{t}\varepsilon_{0}(V_{\mathrm{tg}}-V_{\mathrm{to}})}{d_{t}}$ (ε_b_ and ε_t_: dielectric constant of bottom and top hBN ∼ 3.6; ε_0_: permittivity of air; *e*: charge of electron; *d*_b_ and *d*_t_: thickness of bottom (30 nm) and top hBN (25 nm); and *V*_bo_ and *V*_to_ are the bottom and top gate voltages of CNP at zero magnetic field). From the Landau fan diagram of *R*_*xx*_ and *R*_*yx*_, we find the moiré superlattice carrier density *n*_s_ = 4.65 × 10^12^, 4.72 × 10^12^, and 4.80 × 10^12^ cm^–2^ corresponding to the twist angle of 1.38, 1.41*,* and 1.44*°* using equation $n_{s}=8\theta^{2}/\sqrt{3}a^{2}$ (*a* = 0.234 nm), see the Supplementary Section 1 for more information. AMI 9–1–1 vector magnet is used to study magnetic field direction dependence on *R*_*yx*_. For d*V*/d*I* measurements, an AC excitation voltage of ∼0.1 V rms is applied using a lock-in amplifier, and dc bias voltages is applied using a Yokogawa voltage source meter through a 100 MΩ resistor.

### Analysis of Hall Data and Fittings

We calculated Hall carrier density using equation, $n_{H}=1/e\times \frac{dR_{xy}}{dB_{z}}$ , (here, *e* is elementary charge); the $\frac{dR_{xy}}{dB_{z}}$ is calculated at low *B*_*z*_ range: −0.1 T ≤ *B*_*z*_ ≤ 0.1 T. The high *B*_*z*_ range: −0.5 T ≤ *B*_*z*_ ≤ 0.5 T fitted data are shown in SI Figure S3. The amplitude of *R*_*xy*_ slope w.r.t. *B*_*z*_ shown in SI Figure S4 is calculated by subtracting linear slope of *R*_*xy*_ vs *B*_*z*_ from $\frac{dR_{xy}}{dB_{z}}$ . To quantify the change in the *v*_F_ with displacement field, we estimate Fermi energy *E*_F_ by employing a single-particle equation for the MLG LL_s_ spectrum, *E*_F_ = sgn­(*L*_n_)*v*_F_√(2*eℏ*|*L*_n_| × *B*); where *L*_n_ is Landau level index, *v*_F_ is Fermi velocity of the monolayer Dirac cone, *e* is the elementary charge, and *ℏ* is reduced Planck’s constant. First, we calculated *E*_F_ for LL_1_ at *D* = 0 V/nm as ∼−8 meV at *v* ∼ −0.45, ∼−4 meV at *v* ∼ −0.10, and ∼11 meV at *v* ∼ 0.70 by using *v*_F_ = 10^6^ m/s, see SI Figure S5 for more details. Since *E*_F_ is constant for a given LL, the change in curvature is used to estimate the *v*_F_ change with the displacement field. The *R*_*xy*_ jump amplitude as a function of dc bias current is fitted to the Curie Bloch equation using nonlinear least-squares fitting method.

### Josephson Junction Analysis

The phase space of JJ as a function of the top gate and the back gate is shown in SI Figure S10a. The carrier density (*n*_*j*_) and displacement field (*D*_*j*_) across JJ are calculated using electrostatic equations $n_{j}=\frac{\varepsilon_{b}\varepsilon_{0}(V_{\mathrm{bg}}-V_{\mathrm{bo}})}{ed_{b}}$ and $D_{j}=\frac{\varepsilon_{b}\varepsilon_{0}(V_{\mathrm{bg}}-V_{\mathrm{bo}})}{d_{b}}$ (ε_b_ dielectric constant of bottom ∼3.6; ε_0_: permittivity of air; *e*: charge of electron; *d*_b_: thickness of bottom (30 nm); *V*_bo_ are the bottom gate voltages of CNP at zero magnetic field). The moiré filling factor *v*_*j*_ of JJ is calculated using the equation *v*_*j*_ = *n*_*j*_/*n*_s_ corresponding to *n*_s_ = 4.80 × 10^12^ cm^–2^. The diode effect at *B*_*z*_ = 0*T* is measured using 1 nA ac excitation and switching dc bias current between superconducting critical current *I*_C_ (d*V*/d*I* = 0 Ω) and −*I*_C_. The Curie Bloch equation is fitted to the η and resistance using the nonlinear least-squares fitting method.

## Supplementary Material



## Data Availability

The data supporting the findings in this paper are available from the corresponding author on reasonable request.

## Notes

Stacking and fabrication of device; optical images of stacking and final device; transport characterization of Hall bar devices; Landau Fan diagram of three Hall bar devices; displacement field dependence of renormalize Hall density; evolution of jumps in *R*_*xy*_ as a function of *v* and *D*; line cuts of *R*_*xy*_ for different directions of B; evolution of superconductivity with twist angle and d*V*/d*I* characteristics of SC state; phase space of JJ and temperature dependence of Fraunhofer asymmetry; Fraunhofer pattern measurements at different fillings; transport characteristics of devices with 1.3 and 1.5° twist angles; and Landau fan diagram and IV characteristics of devices with 1.3 and 1.5° twist angles (PDF)
