# Supplementary material for: Gate-Tunable Orbital Magnetism and Competing Superconductivity in Twisted Trilayer Graphene Josephson Junctions
Source: ACS Appl Mater Interfaces. 2025 Dec 8;17(51):69784–94. doi: 10.1021/acsami.5c15822 (PMC12754749; doi:10.1021/acsami.5c15822)
Supplement: Supplementary file 1 [file am5c15822_si_001.pdf]

Supporting Information

Gate-Tunable Orbital Magnetism and Competing Superconductivity in Twisted Trilayer Graphene Josephson Junctions

Vishal Bhardwaj[1†], Lekshmi Rajagopal[1†], Lorenzo Arici[1], Matan Bocarsly[1], Alexey Ilin[1], Gal Shavit[2], Kenji Watanabe[3], Takashi Taniguchi[4], Yuval Oreg[1], Tobias Holder[5], Yuval Ronen[1*]

[1]Department of Condensed Matter Physics, Weizmann Institute of Science, Rehovot 7610001, Israel

[2]Department of Physics and Institute for Quantum Information and Matter, California Institute of Technology, Pasadena, California 91125, USA

[3]Research Center for Functional Materials, National Institute for Materials Science, Tsukuba 305-0044, Japan

[4]International Center for Materials Nanoarchitectonics, National Institute for Materials Science, Tsukuba 305-0044, Japan

[5]School of Physics and Astronomy, Tel Aviv University, Tel Aviv 69978, Israel

[†]These authors contributed equally to this work
*yuval.ronen@weizmann.ac.il

1. Stacking and fabrication of device

The stacks are prepared using flakes exfoliated on Si/SiO_2 substrates. The dome shaped stamps are prepared using polydimethylsiloxane (PDMS) and 6% (by weight) polycarbonate (PC) solution dissolved in chloroform. A big monolayer of graphene around 100×30um is identified using optical microscope and further confirm by analyzing 2D peak of Raman spectra (532nm laser of Witec Raman system). Raman 1064nm laser is used to cut the monolayer into 3 pieces of ~30um length. The hBNs are also exfoliated on Si/SiO_2 and clean areas are identified using optical microscope. The stack is prepared using commercial transfer stage. The top hBN of thickness ~ 25nm is picked at 100° C by Z movement of the stamps. The first graphene layer is picked at 40° C also using Z movement of the stamp. The stage is rotated to around +1.45° and second layer is also picked at same parameters. Subsequently the stage is rotated to -1.45° and third layer is picked. The bottom hBN (~30nm) is picked at 50° C using Z movement of stamps, the stamp wavefront is moved away from stack area and the stage temperature is raised to 180° C to melt the PC with stack on chip. The stack is cleaned using chloroform to remove PC residues. The stack is annealed at 350° C for 2hr in vacuum furnace with base pressure of 5×10^{-8} Torr to release strain and remove any residues on top of stack. The flake thickness is measured, and the topography of stack is cleaned using Bruker AFM. See fig. S1 (a) for a 100X resolution picture of the stack before nanofabrication. The Jeol JBX 9300FS e-beam lithography is used to prepare Hall bar geometry. Double layer PMMA is used in every step of nano fabrication. In the first step, metal top gate is deposited with Cr (5nm) Au (20nm) using e-gun evaporator. In the second step, stack etching is performed using CHF_3/O_2 gas mixture in reactive ion etching machine. Metal bridges (Ti (25nm)/Au (250nm)) are prepared to avoid shorting of top gate leads to the graphene at the edges of stack. The edge contacts to the graphene are made by first etching stack using CHF_3/O_2 gas and subsequently depositing Cr (2nm)/ Au (65nm) metals on 15° angle using e-gun evaporator while rotating the sample holder. The final device consists of three Hall bar devices A, B and C. There are two Josephson junctions, first between device A and B of width

~200nm (JJ1) and second between device B and C of width ~100nm (JJ2). See fig. S1 (b) for the image of the final device, after fabrication process.

The transport properties are measured in LD400 Bluefors dilution refrigerator. The fridge has thermo-coax lines and low pass RC and RF filters. It has 9-1-1 magnet with base temperature of 7mK. Standard 830SRS and 865SRS lock in amplifiers are used to measure transport properties. Femto DLPVA-101-F-D room temperature voltage amplifiers (1TΩ input impedance) are used before feeding signal to lock in amplifiers. Keithley 2400 voltage source meters are used for controlling voltages of gates. For dV/dI characteristics, Yokogawa is used as dc source meter and lock in as AC source. The carrier density (n) and displacement field (D) are calculated using electrostatics equations $n = \frac{\varepsilon_b \varepsilon_0 V_{bg}}{e d_b} + \frac{\varepsilon_t \varepsilon_0 V_{tg}}{e d_t}$ and $D = \frac{\varepsilon_b \varepsilon_0 V_{bg}}{d_b}$ - $\frac{\varepsilon_t \varepsilon_0 V_{tg}}{d_t}$ respectively. Here, ε_0 is the dielectric constant of vacuum and is equal to $8.854 \times 10^{-12}\ \mathrm{CV^{-1}m^{-1}}$, $\varepsilon_t(\varepsilon_b)$ are the relative dielectric constant of the top (bottom) hBN ~ 3.9, $d_t(d_b)$ are thickness of the top hBN (Si/SiO_2 +bottom hBN) which we estimated to be 25 nm (285nm +30 nm), $V_{tg}(V_{bg})$ are the voltages of top (bottom) gates. The moiré super lattice carrier density (n_s) for device A, B and C are $4.4 \times 10^{12}, 4.65 \times 10^{12}$and $4.8 \times 10^{12} cm^{-2}$ respectively. The twist angles are estimated using equation $n_s = 8\theta^2/\sqrt{3}\ a^2$ (a=0.234nm) and are found to be 1.38°, 1.41°, and 1.44° for A, B and C device, respectively.

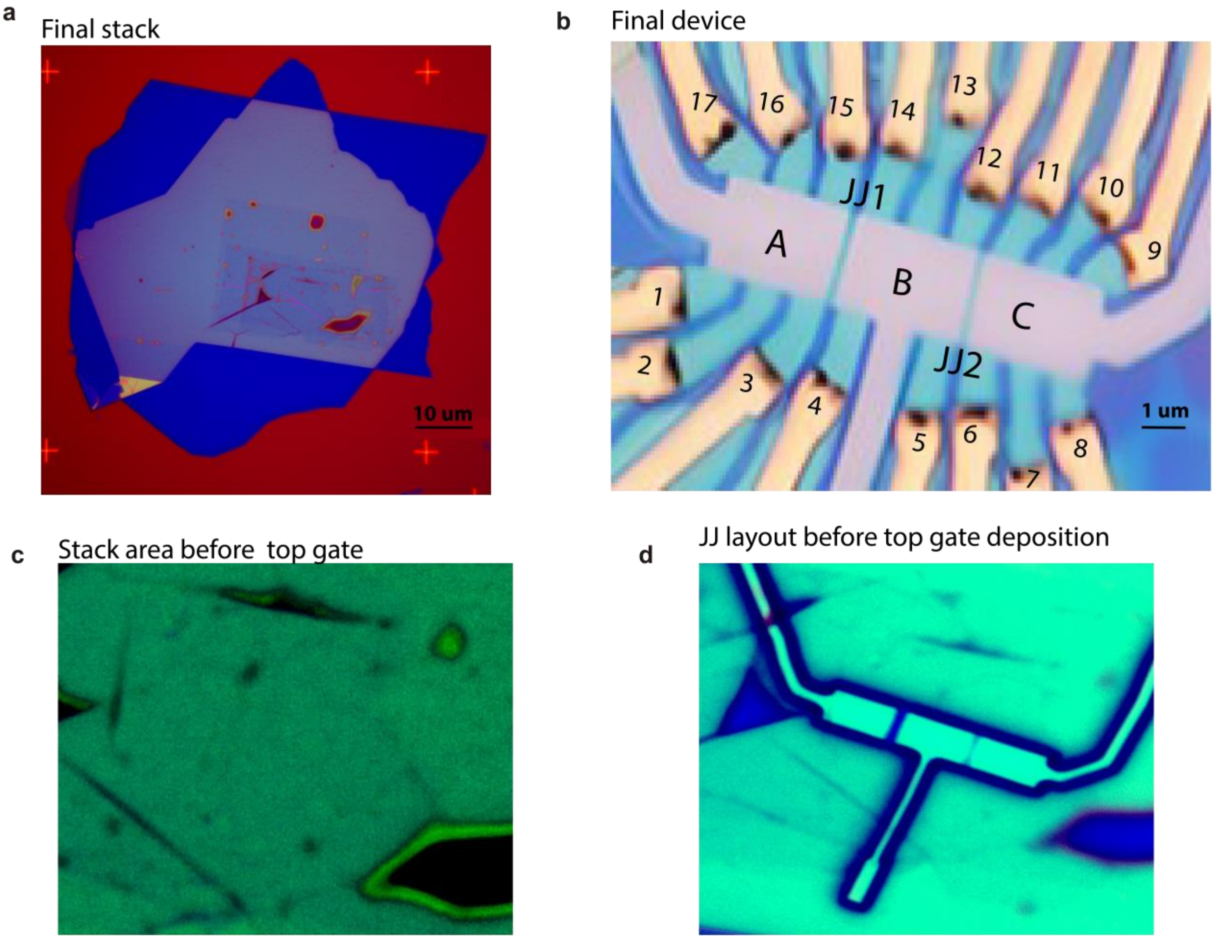

Figure S1: (a) High-magnification (100×) optical micrograph of the TTG stack prior to nanofabrication; the region later used for device definition is outlined. Scale bar, 10 µm. **(b)** Optical micrograph of the completed structure showing three

top-gated sections (A–C) and contact numbering. Two electrostatically defined Josephson junctions (JJ1 and JJ2) have weak-link lengths of ~200 nm and ~100 nm, respectively. The Hall-bar width is ~2 μm. Scale bar, 1 μm. **(c)** Zoomed pre-gate image of the area where devices A–C were patterned, highlighting the bubble-free region selected for transport. **(d)** Optical image of the developed resist prior to top-gate metal deposition, showing the patterned gates for devices A–C and the junction footprints.

2. Transport characterization of hall bar devices:

When we dope, the electrons (holes) to the conduction (valence) bands of the TTG are distributed between the flat bands and ML Dirac cone. Most of the charge carriers are filled in the flat bands due to high density of state (DOS) and the rest goes to ML Dirac cone[1,2]. The mini-Brillouin zone of TTG has four-fold degenerate bands with flavors $K, K', \uparrow, \downarrow$. When we start doping electrons(holes) from *ν*=0, all four flavors start filling the conduction (valence) bands equally, and hence a linear (unity) slope between Hall carrier density (n_H) and *ν* is expected in the vicinity of CNP[3]. At *ν*=±1 (±3), the symmetry of four-fold degenerate bands breaks and out of four anyone (three) flavor is filled completely which result in resets towards zero. Similarly at *ν*=±2, due to symmetry breaking of spin and valley isospin degenerate bands, two flavors of either spin or valley isospin are filled completely, and a reset is observed. At *ν*=±4 all four flavors are filled and Fermi energy (E_F) exits the flat bands and we observe reset toward zero again. This symmetry breaking of degenerate bands results in Chern insulator states at integer *ν*, as seen in Landau fan diagrams of R_{xx} and R_{xy} at D=0V/nm (see Fig S2).

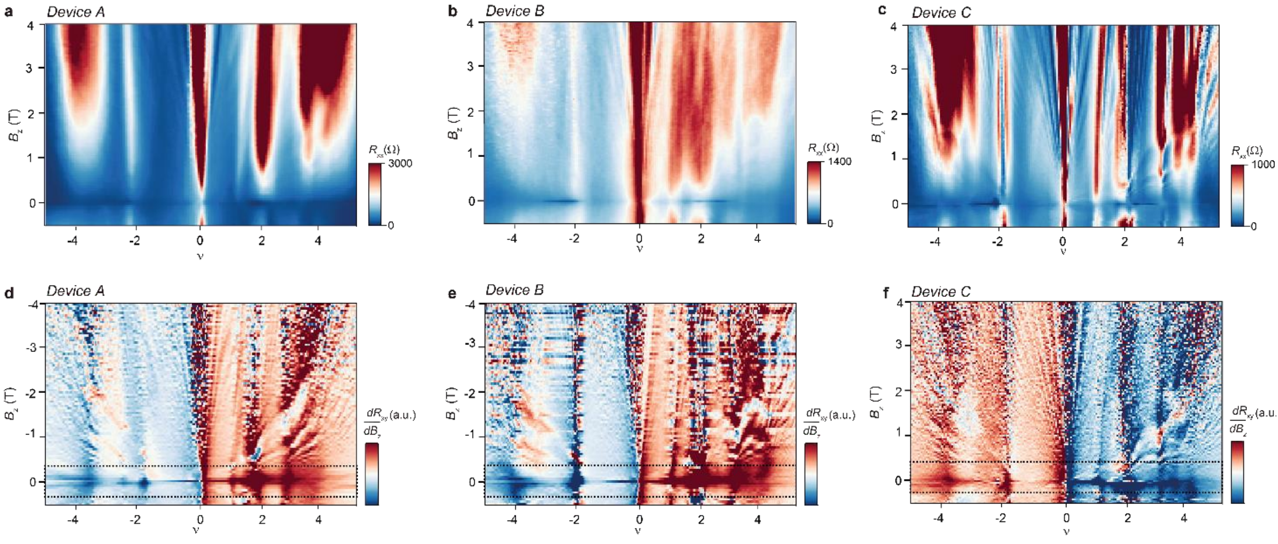

Figure S2: The Landau fan diagrams of R_{xx} and R_{xy}. Measurements are taken at 15mK and D=0V/nm for device **(a)** A (contacts 2-3) **(b)** B (contacts 14-13) and **(c)** C (contacts 6-7). The Landau fan diagram of $\frac{dR_{xy}}{dB_z}$ measured at 15mK and D=0V/nm for device **(a)** A (contacts 15-3) **(b)** B (contacts 14-4) and **(c)** C (contacts 11-6). Dashed black boxes show the slope change in $\frac{dR_{xy}}{dB_z}$ around B_z =0T.

We calculated the normalized Hall carrier density using equation $\nu_H = 1/(e * \frac{dR_{xy}}{dB_z} * n_s)$; (here, *e* is elementary charge, n_s moiré superlattice density), the $\frac{dR_{xy}}{dB_z}$ is calculated at low B_z range (-0.1T ≤B_z ≤0.1T) and high B_z range (-

0.5T ≤B_z ≤0.5T) around B_z= 0T. The variation of quantity $(\nu_H - \nu)$ with ν shed light on the evolution of TTG DOS with varying E_F[4,5]. Fig. S3(a) and (b) shows the $(\nu_H - \nu)$ *vs* ν plot for high B (red) and low B (blue) ranges at D=0V/nm and 0.6V/nm, respectively. The integer values of $(\nu_H - \nu)$ are expected at integer ν corresponding to symmetry breaking and we observe this condition at ν=+1, ±2, +3 and ±4, see red curve in fig. S3 (a). However, in addition to that a pronounced kink is observed in the vicinity of CNP towards hole doping and two small kinks are observed just before ν=+2 and -2 as shown by black arrows in fig. S3 (a). These kinks are also associated with the symmetry breaking of flavors and presence of isospin ferromagnetic state[5,6]. The behavior of sample at high displacement field looks completely different, as seen in fig. S3 (b). The kinks near the CNP vanishes and strong van hove singularities are observed at ν=±2 and ν=±3, i.e. on the boundary of superconductivity region. This signifies the strengthening of superconductivity at high displacement field in 2≤ $|\nu|$ ≤3[4]. The Landau fan diagrams of $\frac{dR_{xy}}{dB_z}$ at high displacement fields (0.6V/nm) is shown in fig. S3(c)-(d) for devices A and C respectively. The jumps in R_{xy} around B_z=0 in both devices have vanished. An interesting note is that in Device C ($\theta = 1.44°$), ν=3 becomes visible which is absent in device A ($\theta = 1.38°$).

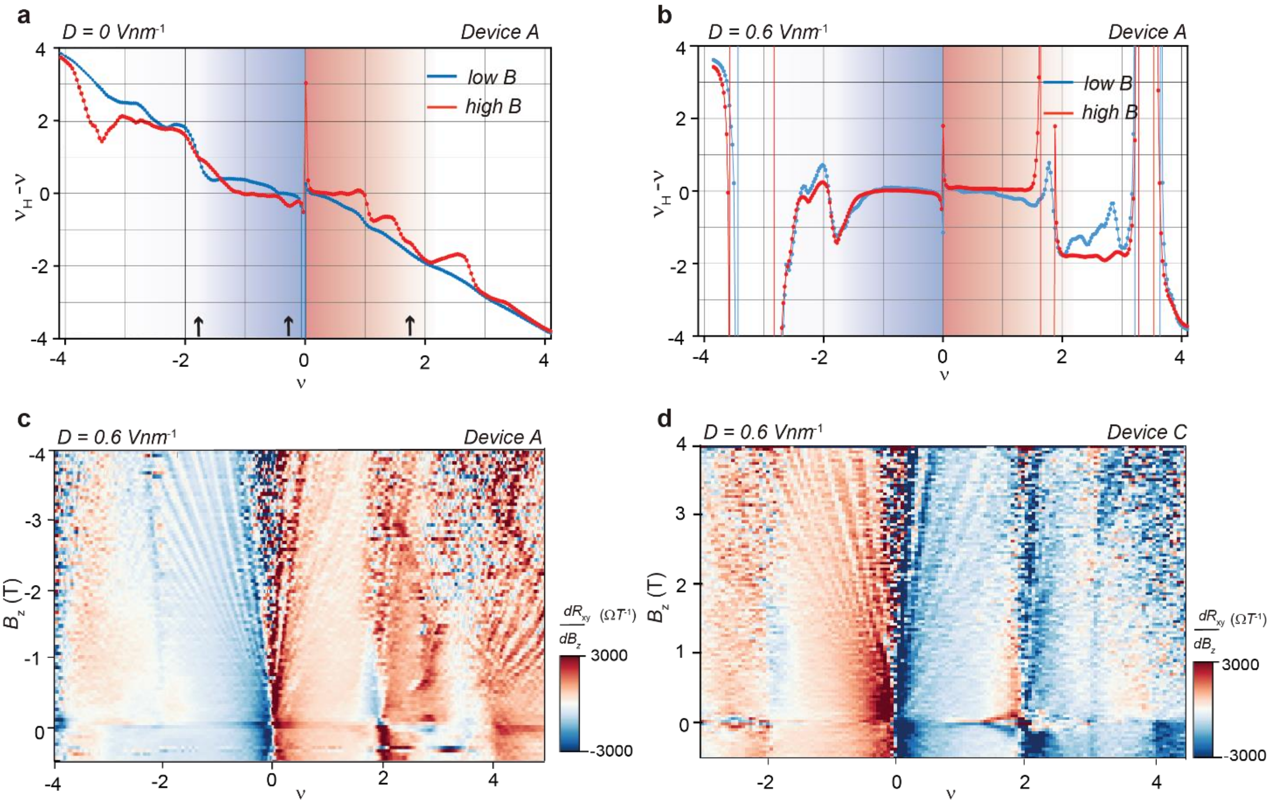

Figure S3: The moiré filing factor (ν) subtracted from renormalize Hall density $\nu_H = B_z / e\rho_{xy} n_s$ vs ν plots at low B_z (-0.1T ≤B_z ≤0.1T) and high B_z (-0.5T ≤B_z ≤0.5T) shown by blue and red colors, respectively at **(a)** D=0V/nm and **(b)** D=0.6V/nm. Landau level fan diagram of $\frac{dR_{xy}}{dB_z}$ at D=0.6V/nm for device **(c)** A and **(d)** C.

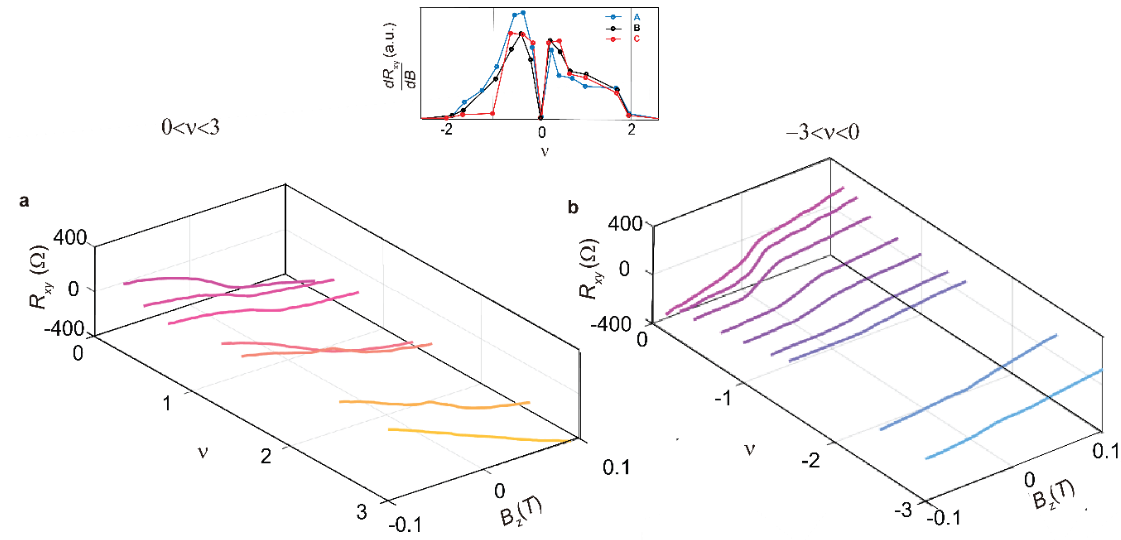

Figure S4: Evolution of jumps in R_{xy} as a function of *ν* for electron and hole-doped regions. Line cuts of R_{xy} vs B_z for **(a)** electron and **(b)** hole doping at D=0V/nm for device A. Inset shows the variation of $\frac{dR_{xy}}{dB_z}$ amplitude around B_z=0 extracted after subtracting the linear slope of R_{xy} at high B_z as a function of filling factor *ν* for all three devices. Maxima is obtained in vicinity to CNP on hole doping side.

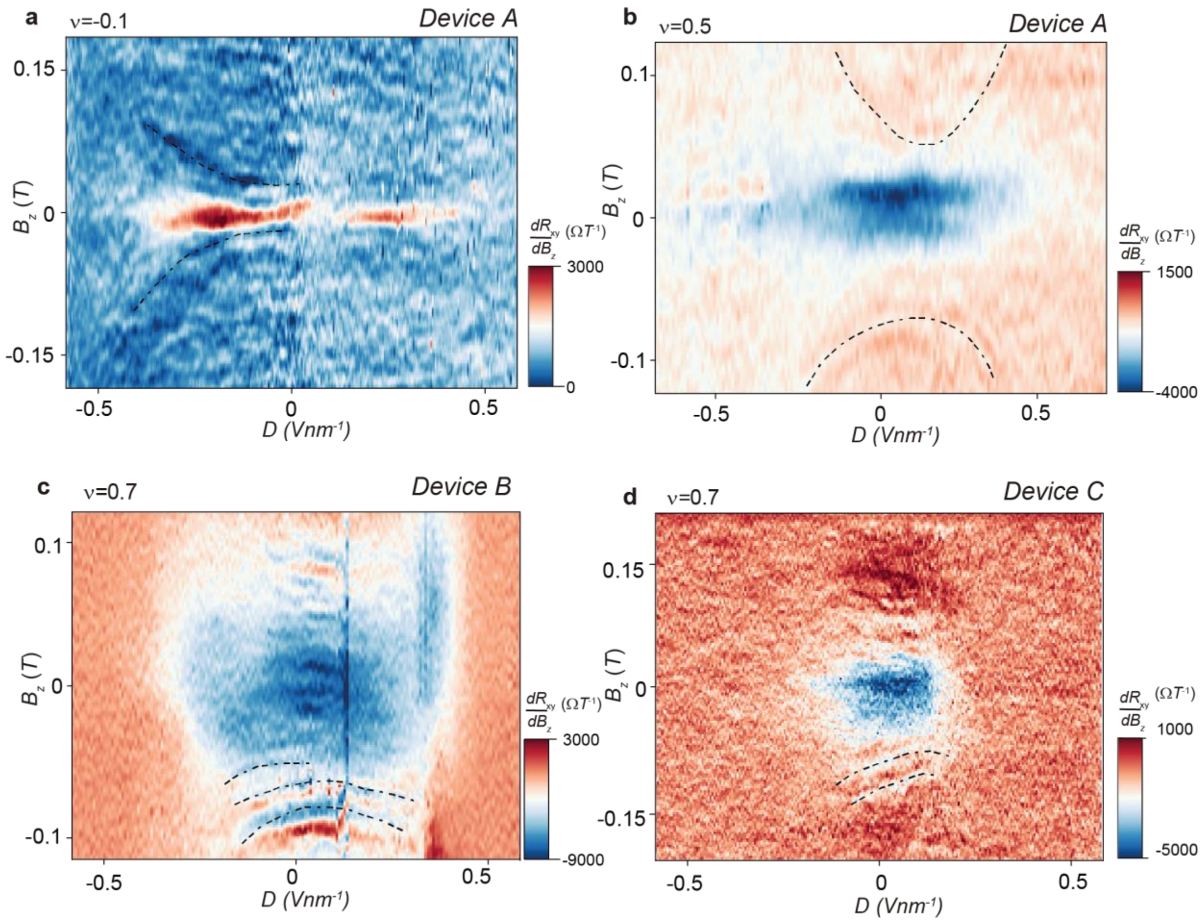

Figure S5: The 2D plots of $\frac{dR_{xy}}{dB_z}$ for B_z *vs* D at fixed **(a)** ν =-0.1 (device A), **(b)** ν =0.5 (device A) **(c)** ν=0.7 (device B) and **(d)** ν=0.7 (device C).

Supplementary Fig. S4 compiles $R_{xy}(B_z)$at $D = 0$V/nm on both sides of charge neutrality. To quantify the near-zero-field feature, we remove the linear high-$|B_z|$ background (fit in a symmetric high-field window) and evaluate $|dR_{xy}/dB_z|$within a small symmetric window about $B_z = 0$. The result is robust to moderate changes in either window and repeatable across sweeps. Within $|\nu| \lesssim 0.7$the curves share a common line shape with a sharp slope jump; the amplitude peaks near $\nu \approx -0.45$and shows a secondary maximum near $+0.70$, then decreases as $|\nu|$increases.

The TTG band structure consist of flat bands of TBG at $\sqrt{2}$ times the twist angle and a bystander monolayer Dirac cone. The mirror symmetric configuration of TTG means the localization of wavefunction on top/bottom layers can be tuned by the application of displacement field. At D=0V/nm, flat bands and Dirac cone are decoupled, however with application of D the flat bands and Dirac cone start hybridizing. This hybridization can result in rich physics. Here we are utilizing the ML Dirac cone Landau levels and their hybridization as a probe to estimate the E_F of flat bands[1,2]. We observe large value of $\left|\frac{dR_{xy}}{dB_z}\right|$ near by $B_z = 0T$ due to orbital magnetism (OM) which disappear with the increase in hybridization (D). The parabolic shaped curves in the figures represent the Landau levels (LL_s) of monolayer graphene at constant Fermi energy (E_F) defined by ν. The most prominent feature is the upward curvature of these curves with increasing *D*, which results from the hybridization between the MLG Dirac cone and the flat bands[1]. As *D*, and therefore hybridization increases, the slope of the MLG Dirac cone, *i.e.* Fermi velocity, $v_F = dE/dk$ decreases. Since E_F is fixed, the LL_s appear at higher *B*. To quantify the change in the v_F, we estimate Fermi energy E_F by employing a single-particle equation for the MLG LL_s spectrum, $E_F = sgn(L_n)v_F\sqrt{(2e\hbar\,|L_n| \times B)}$; where L_n is Landau level index, v_F is Fermi velocity of the monolayer Dirac cone, *e* is the elementary charge, and ħ is reduced Planck's constant. We estimated E_F~-8meV for LL_1 and ~-9meV for LL_2 at ν ~-0.45 (see Fig 2 (a) main file) and E_F~-4meV at ν ~-0.10 we, see fig. S5(a). As we tune E_F to electron doped ν~0.70 we estimated E_F~11meV for LL_1 and E_F~13meV for LL_2, see Fig 2 (b) main file. These values of E_F at corresponding ν matches exactly with the estimation made using squid on tip scanning experiments[1]. The upward curvature of LL_s lines with D indicate the hybridization of monolayer Dirac cone with flat bands, with the increase in D the slope of dE/dk decreases and hence v_F decreases, since the E_F is fixed the LL_s are observed at higher B_z in higher D. The estimated v_F change at ν=-0.10 and -0.45 is ~67% and 45% for D change from 0 to 0.5V/nm. At ν=0.70 v_F change by ~15% for D change from 0 to 0.25V/nm.

As the filling detunes from charge neutrality at $D = 0$, the Hartree charging self-energy increases and preferentially raises/reshapes the AAA-localized flat-band states, broadening the flat bands (larger W) and lowering the effective interaction ratio U/W. This redistribution transfers spectral weight and carriers toward the more dispersive monolayer-Dirac sector, thereby diluting the Berry-curvature concentration associated with the flat bands near E_F. Consistent with this picture, the near-zero-field Hall-slope jump $|dR_{xy}/dB_z|$compiled in Fig. S4 decreases as $|\nu|$increases. Raising $|D|$ breaks mirror symmetry and hybridizes the Dirac and flat-band sectors, which we quantify *via* the reduction of the Dirac $v_F$extracted from the MLG-LL curvature (SI Fig. S5 and main-text Fig. 2). Enhanced hybridization further disperses Berry curvature and correlates with the suppression of the near-zero-field jump (Fig. S5). We do not fit a microscopic model here; rather, the observed ν- and D-trends are qualitatively consistent with the band-renormalization picture established in Ref. 1. More broadly, broader and more hybridized bands are generally less conducive to interaction-driven

symmetry-broken states, providing a natural context for the fade-out of the anomaly away from the low-| ν |, low-| D | regime.

Fig. S6(a) and (c) shows the R_{xy} jumps in forward and backward sweeping of magnetic field at ν=-0.45 and ν=0.7 respectively at D=0 V/nm. We note very small hysteresis in these plots and the presence of a clear jump. The observed trend in these measurements is the opposite and can come from minor drifts or delays in superconducting magnets. To rule out this these scans are taken at extremely slow scan rate of 0.1 G/min and we also measured the sample in two different fridges one with 18T magnet and one with 9-1-1 magnet, yet we still observed small shifts in the data, which further indicates that the observed effects are intrinsic rather than due to instrumental drift.

The corresponding $R_{xy} - B_z$ sweeps at higher displacement, D=0.6 V/nm shows that these jumps have vanished, see fig. S7 (b) and (d). We explicitly show the dependence of R_{xy} jumps in 0° to 90° direction of the applied magnetic field $(B_x^2 + B_z^2)^{1/2}$ for device B and C respectively in fig. S7 (a) and (b). The R_{xy} jumps are present in all devices but are most prominent in device A (main fig. 3 and fig. S4) and decreases as we move to twist angles closer to the magic angle. We show the linecuts of dV/dI characteristics of the JJ2 taken at B_Z=0G, with configuration S|X|S, where S corresponds to ν~2.7 and X corresponds to ν_j, which is varied from ~-3 to 2.5. We see asymmetricity in the dV/dI w.r.t I_{dc} in ν_j close to the CNP, but this dies off at higher ν_j, see fig. S8(a). Figure S8 (b) shows the temperature dependence of dV/dI characteristics across the JJ2 at $\nu_j = -0.45$. The curve becomes approximately symmetric at 650mK. We characterize the SC in our near magic angle device, Device C. From R_{xx}-T plot of Device C, T_C ~ 1.3 K estimated corresponding to the 90% drop in resistance, see fig. S8 (c). Figure S8 (d) shows the line cut of the transverse resistance R_{xy} measured using contacts 12–5 on device B at $D = 0.6\ V/nm$ and $T = 20\ mK$, i.e., the same contacts used to define the JJ across B and C. A clear zero-resistance plateau is observed between filling factors $\pm 2 < \nu < \pm 3$, highlighted in red in the attached figure. This provides direct evidence that the region of device B participating in the JJ is indeed superconducting under the operating gate conditions.

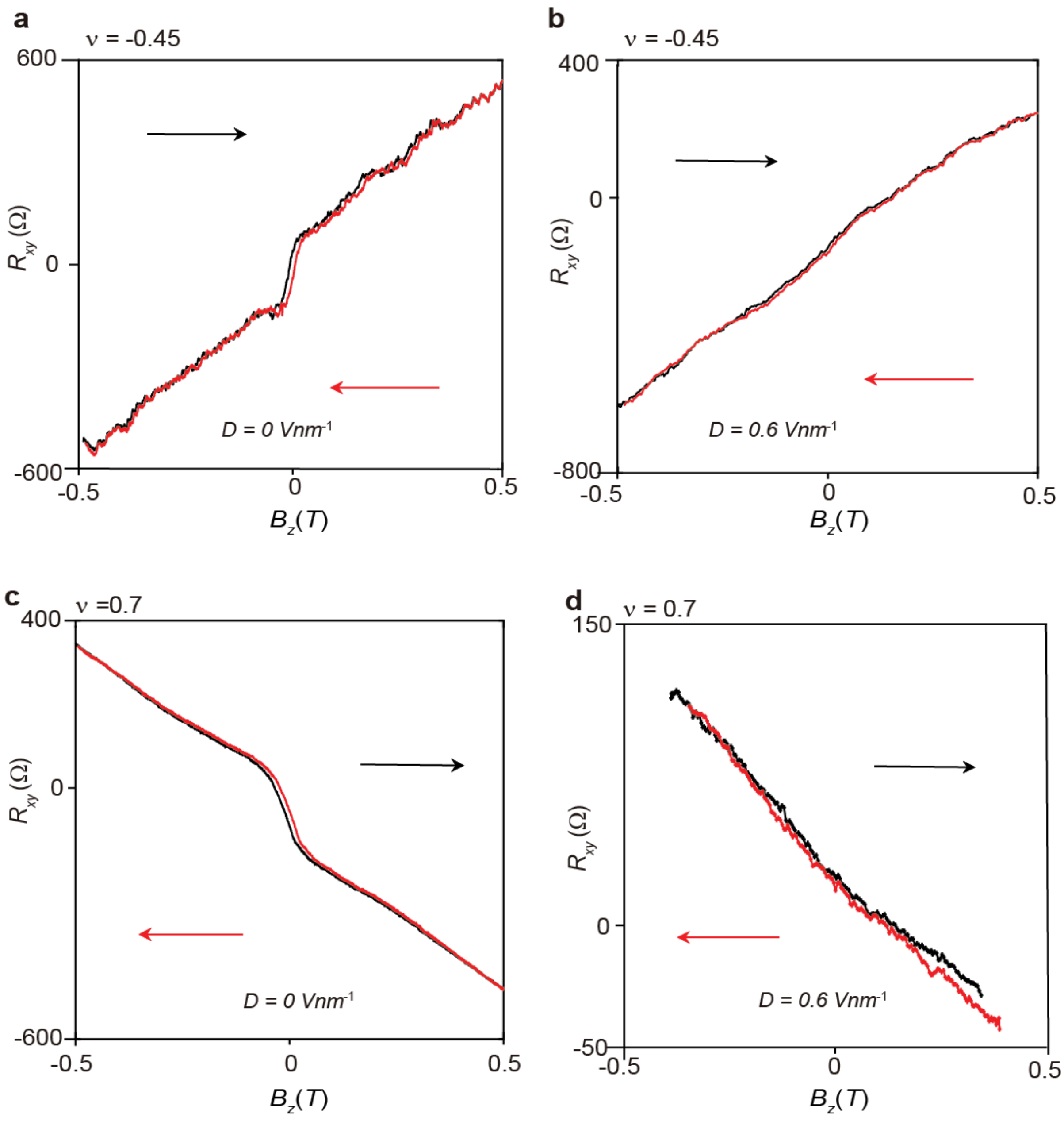

Figure S6: Line cuts of R_{xy} for B_z sweep directions -0.5T to 0.5T (black) and 0.5T to -0.5T (red) at **(a)** v = -0.45, D=0V/nm, **(b)** v = -0.45 and D=0.6V/nm; **(c)** v =0.70, D=0V/nm and **(b)** v = 0.70and D=0.6V/nm for device A.

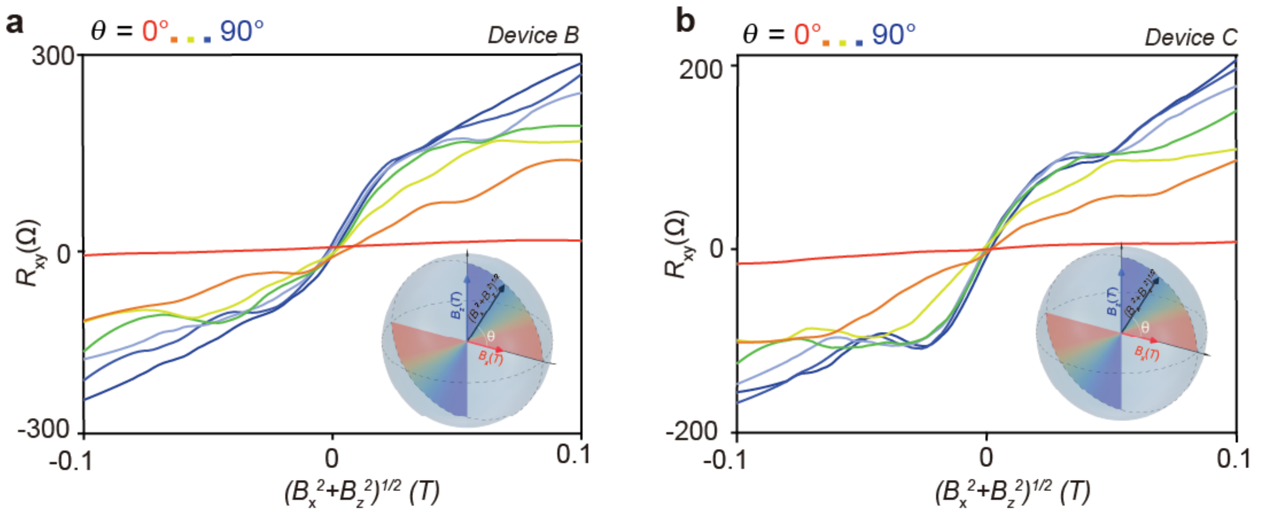

Figure S7: The R_{xy} vs $B = (B_x^2 + B_z^2)^{1/2}$ at varying θ between sample and B from 90° (blue) to 0°(red) in steps of 15° measured at 15mk for **(a)** device A and **(b)** device B.

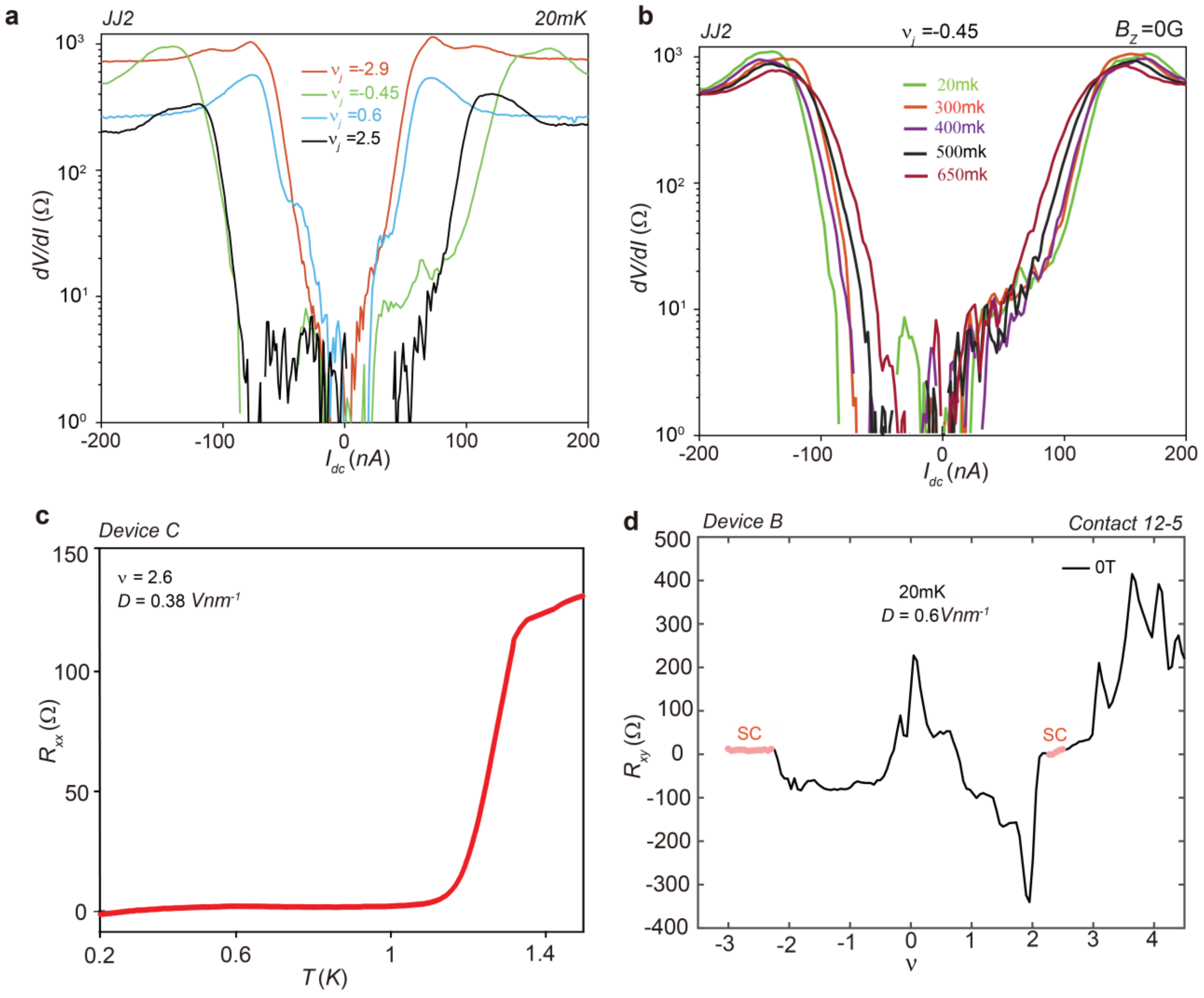

Figure S8: **(a)** The dV/dI *vs* I_{dc} characteristics for SC state tuned to ν~2.7 across JJ2 and weak link tuned to different ν_j shown in figure legends. **(b)** Temperature dependence of dV/dI *vs* I_{dc} characteristics ν_j ~-0.45 across JJ2. **(c)** The R_{xx}-

T plot of device C measured at $B_z = 0T$, v=2.35 and D=0.42V/nm. **(d)** Line cut of R_{xy} as a function of v for device B (contacts 12-5, $D = 0.6\,V/nm$ and $T = 20\,mK$). A clear zero resistance plateau is observed between ±2<v<±3, highlighted by red, confirming the presence of superconductivity in the portion of device B used to define the junction.

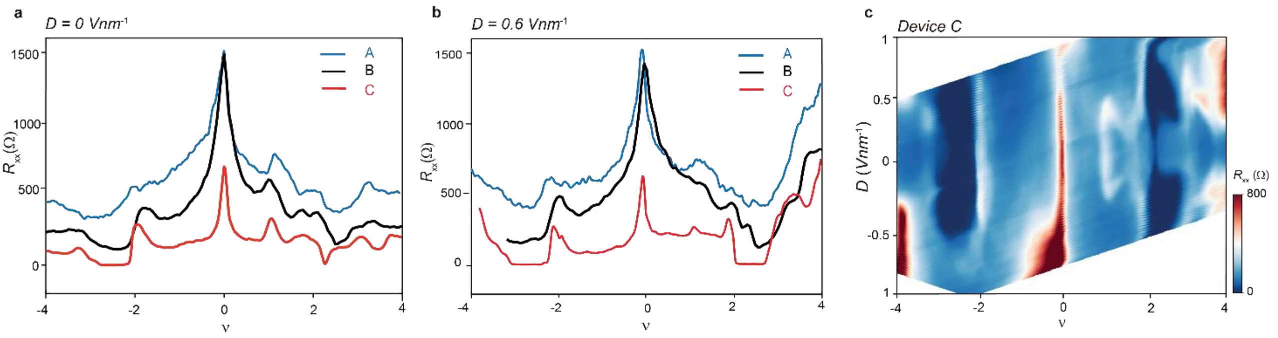

Figure S9: Evolution of superconductivity with twist angle. Line cuts of R_{xx} vs v for all three devices at T=20mk and D = **(a)** 0V/nm and **(b)** 0.6V/nm. **(c)** D vs v phase space of R_{xx} for device C, at T=20mK.

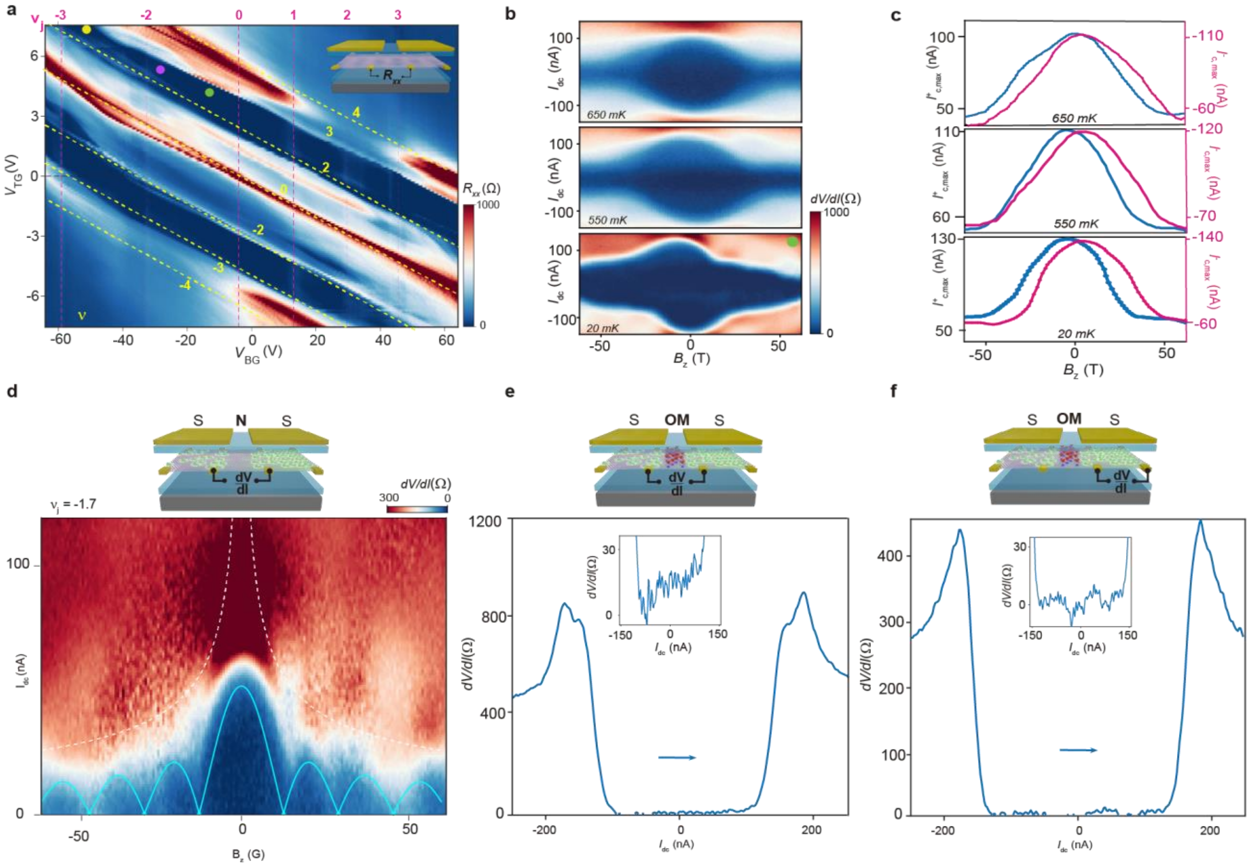

Figure S10: Phase space of Josephson junction and temperature dependence of Fraunhofer asymmetry. (a) Phase space of R_{xx} as a function of top gate (V_{TG}) vs back gate voltage (V_{BG}) across JJ2 at 20mK. Slanted yellow dashed lines indicate the filling factor (v) for the left and right sides of the JJ (dual-gated). Vertical pink dashed lines correspond to the weak link filling factor (v_j) (back-gated only). **(b)** The Fraunhofer pattern measured at v_j~-0.45 forming S|OM|S JJ at

20mK, 500mK and 650mK. **(c)** Line cuts of the maximum critical current extracted from figure b. **(d)** The theoretical fit for the Fraunhofer pattern from our JJ at S|N|S configuration (ν_j ~-1.7). The cyan line marks the fitted node positions using $\Delta B \approx \frac{\pi\phi_0}{4a_0 w^2}$; where ϕ_0 is the magnetic flux quantum, w is the junction width, and a_0 is a geometric factor derived from fitting the interference pattern using Bessel functions[44] . The white line follows the expected lobe amplitude decay $\propto 1/\sqrt{B}$, consistent with ballistic transport in 2D Josephson junctions. **(e)** Asymmetric I–V characteristics across S|OM|S junction indicating TRS breaking in the weak link. **(f)** Symmetric I–V in device C, confirming TRS breaking is localized to the junction. See insets for zoomed curves.

We can switch between superconducting (SC) and non-SC states by alternating I_{dc} between $\pm I_c^-$ on the asymmetric curve of dV/dI at B_z=0 G. Fig. S11(a) shows the dV/dI (Ω) characteristics measured by alternating between I_c^- (dV/dI=0 Ω) and -I_c^- every 20 seconds and plotted over time in the temperature range 20mK to 650mK. The resistance R in the non-SC state decreases with increasing temperature. We fitted the temperature dependence of R to a Curie Bloch law, viz. $R = m(1 - T/T_{OM3})^{\gamma}$, and estimated m= 30.05±1.5Ω, γ ~0.24±0.05 and T_{OM3}~650mK as seen in Fig. S11(b). The exponent of Curie Bloch law is expected to contain information about the magnetic moments, for example for a magnetic state of 2D Ising nature a critical exponent of 0.125 is expected[7,8] . However, in our case we measure the exponent 0.24±0.05 from resistance of the diode-like effect, 0.45±0.05 from the R_{xy} data and 0.6±0.2 from the asymmetry in critical currents. The scaling behaviors are consistent provided the resistance of diode effect is coupled to ~M^2, R_{xy} to ~M^3 and η to ~M^4 [9,10]. However, we believe a microscopic picture is needed to clarify this point.

We show the Fraunhofer patterns in two *e-h* Josephson junction configurations, Fig. S12 (a) S`|OM|S`` and (b) S`|N|S`` in JJ2, here S` and S`` correspond to $h$ and $e$ side SC, respectively. We see that while S`|N|S`` ($\nu_L = -2.5, \nu_j = 1.8,\ \nu_R$~2.7) shows a clear Fraunhofer pattern with clear dips in interference, we do not see a clear pattern in S`|OM|S`` ($\nu_L = -2.4,\ \nu_j = 0.5,\ \nu_R$~2.7) . Moreover, the weak Fraunhofer is also slightly asymmetric about $I_{dc} = 0$, owing to the OM.

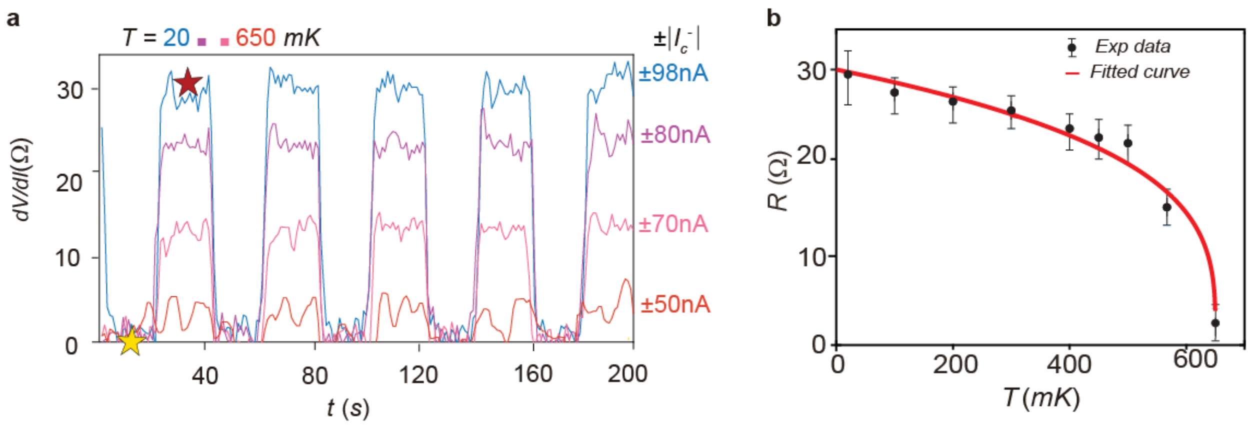

Figure S11: (a)) The dV/dI (Ω) characteristics of S|OM|S JJ measured at B_z=0 G while alternating I_{dc} between I_c^- (dV/dI=0 Ω, SC state) and $-I_c^-$ (non-SC) after every 20 seconds in a temperature range of 20mK to 650mK. **(b)** Resistance of the non-SC state (R) calculated from Fig 4e and plotted as a function of temperature (black data points). The red line corresponds to fits of the Curie Bloch equation $\Delta R = m(1 - T/T_{OM3})^{\gamma}$. The error bars depict the standard deviations in R.

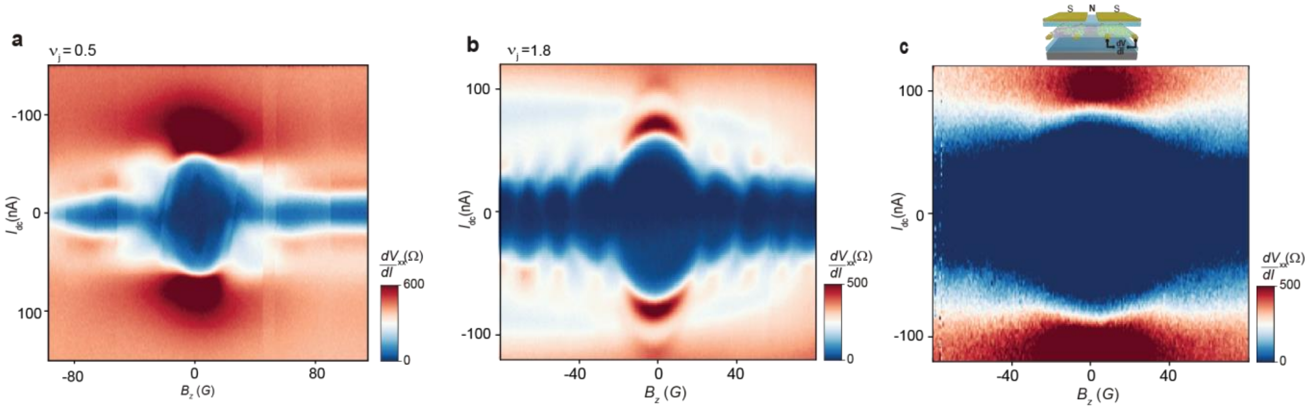

Figure S12: The Fraunhofer pattern measurements at (**a)** The S`|OM|S`` JJ configuration (BG=3.8V, TgL=-4.4, TgR=2.8) the left side of JJ SC state tuned to $v_L \sim -2.5$, right side SC $v_R \sim 2.7$ and weak link tuned $v_j \sim 0.5$. **(b)** The S`|N|S`` JJ configuration (BG=27.6V, TgL=-6.2, TgR=0.7) corresponding to hole side SC on left side (S`) $v_L \sim -2.4$ and electron side SC on right side (S``) $v_R \sim 2.7$ of the JJ with weak link tuned to $v_j \sim 1.8$. **(c)** Hall-bar control on the right side of JJ2. Map of differential resistance dV_{xx}/dIversus out-of-plane field $B_z$and DC bias I_{dc}taken at the same gate settings used in Fig. 4b. The Hall-bar segment is uniformly superconducting (S|S|S): the critical-current envelope narrows smoothly with $|B_z|$and shows no periodic nodes (nodeless $I_c(B_z)$). This confirms the absence of an unintended weak link in the Hall bar; Josephson interference appears only at the lithographic B|C junction. Inset: measurement geometry.

3. Transport characteristics of devices with 1.3° and 1.5° twist angles

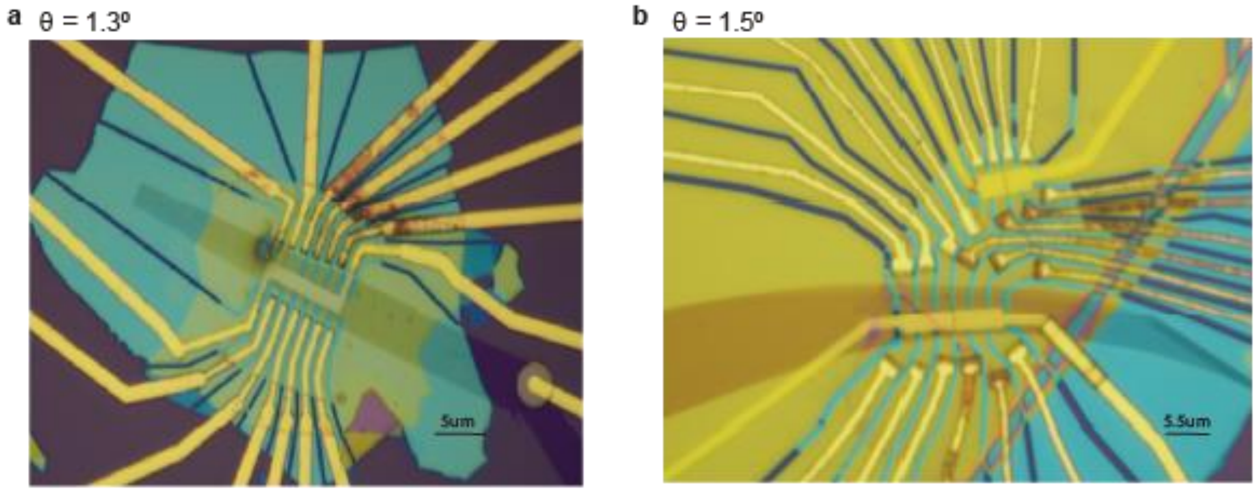

Figure S13: The additional TTG devices with twist angles **(a)** 1.3^0 and **(b)** 1.5^0**.**

We stacked two other samples with twist angle 1.30° and 1.50° to explore OM in region above and below 1.40° regime, see fig. S13. Sample with 1.30° twist angle has top hBN~32nm and bottom hBN~55nm thickness. This sample has

graphite as bottom gate. Sample with twist angle 1.50° has top hBN~60nm and bottom hBN~36 nm. This sample has two different Josephson junction devices, one has graphite as bottom gate and other has Si as bottom gate.

Fig. S14 (a) and (c) shows the Landau fan diagram of $R_{xx}/\square$ and R_{xy} respectively for sample 1.30° at D=0V/nm. We observe correlated insulators at ν=+2, ±4 only in this sample. Interestingly SC is observed only in electron side in this sample. Fig. S14 (b) and (d) shows the Landau Fan of R_{xx} and R_{xy} for sample with twist angle 1.50°, respectively at D=0V/nm. In this sample we observe Chern insulator states at ν=±2, +3 and ±4. A wide pocket of SC is observed on electron doping side in comparison to hole doping. No jumps in R_{xy} are observed in both samples around B_z= 0.

Next, we analyzed the SC in both samples. Fig. S15 (a)-(c) shows the characterization of SC in sample with twist angle 1.30°. Fig. S15 (a) shows the temperature dependence (10mK to 1.2K) of dV/dI vs I_{dc} characteristics. The T_C of ~1.0 K is estimated from the $R_{xx}/\square$ vs T plot, corresponding to the 90% drop in resistance, see Fig. S15 (b). The Ginzburg Landau equation, $B_{c2} = (\varphi_0/2\pi\xi^2{}_{GL})(1-\frac{T}{T_c})$,(where $\varphi_0 = h/2e$ is superconducting flux quantum, ξ_{GL} is coherence length) is fitted to the temperature dependence of B_{c2} taken at 90% drop in R_{xx} at that temperature, see fig. S15 (c). The upper critical field at 0K *i.e* B_{c2} (0) ~ 0.13T is estimated from fitting which corresponds to ξ_{GL} ~50nm. Fig. S15 (d) shows the Fraunhofer pattern measured in e-S| N |h-SC configuration of 1.50° device.

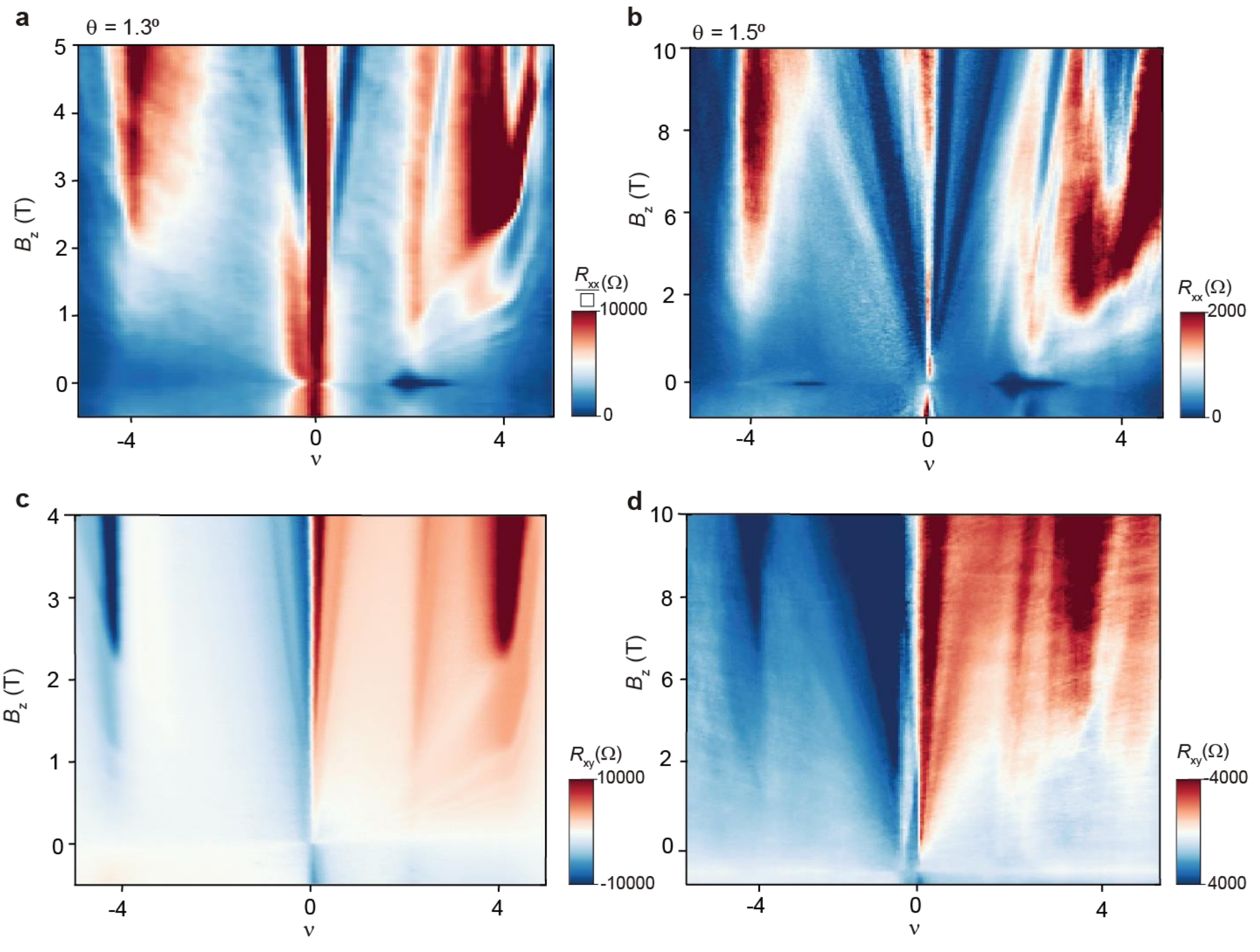

Figure S14: The Landau fan diagram **(a)** of $R_{xx}/\square$ for TTG with twist angle 1.30° and **(b)** of R_{xx} for TTG with 1.50° measured at D=0V/nm and 15mk. The Landau fan diagram of R_{xy} **(c)** for 1.30° TTG device at 300mK and **(d)** for 1.50° TTG device at 15mK and D=0V/nm.

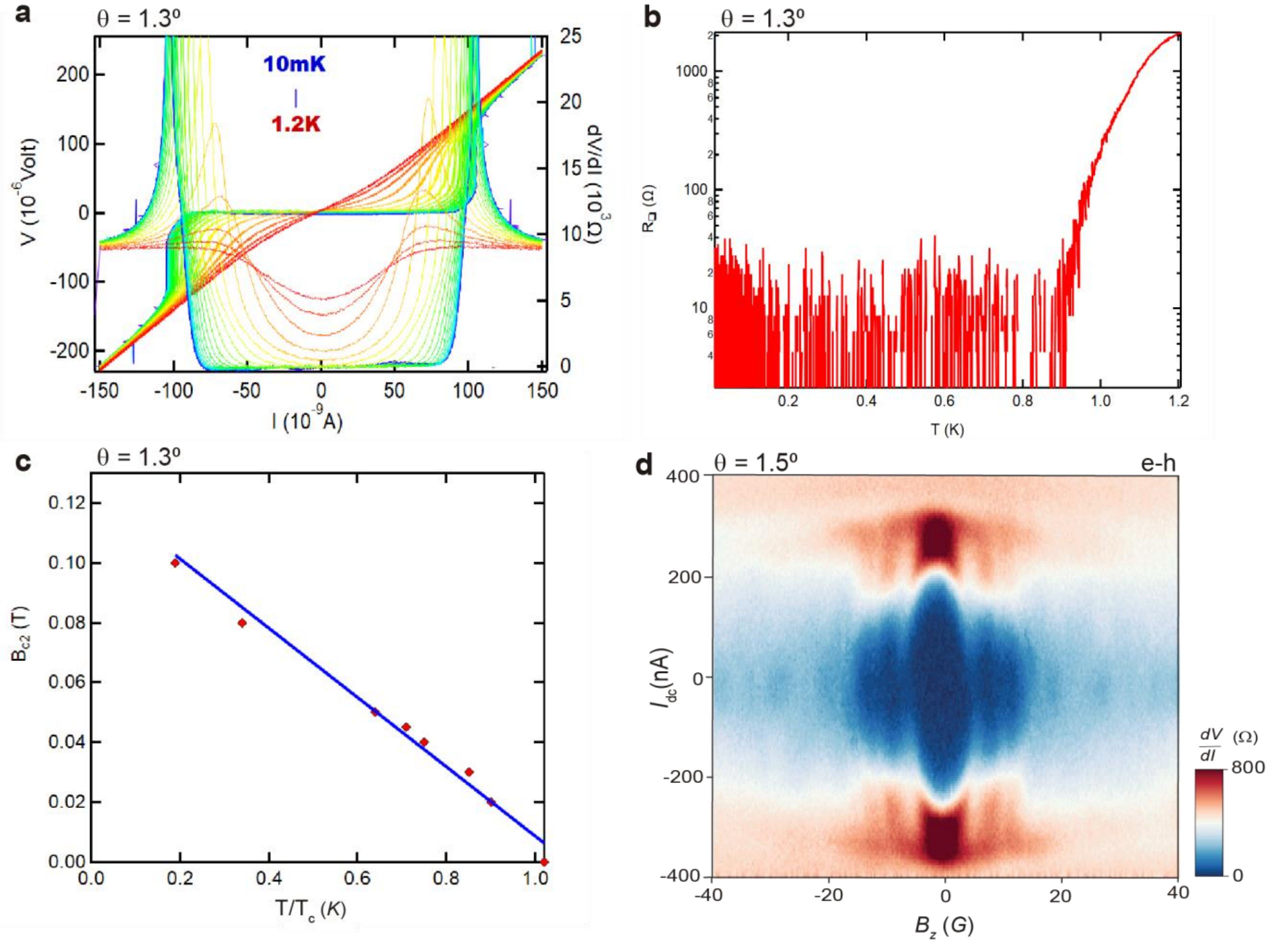

Figure S15: (a) IV characteristics of TTG device twist angle of 1.3^0 as the temperatures are varied from 10mK to 1.2K. **(b)**The $R_{xx}/\square$ vs T curve for the same device with critical temperature T_c=1K. **(c)** The critical magnetic field B_{c2} is plotted as a function of T/T_c and the B_{c2} (0 K) is estimated to be approximately 0.13 T. **(d)** Fraunhofer pattern of e-h doped superconducting pockets for the device with twist angle 1.5^0.

References:

1. Bocarsly, M. *et al.* Imaging Coulomb interactions and migrating Dirac cones in twisted graphene by local quantum oscillations. *arXiv* (2024).

2. Shen, C. *et al.* Dirac spectroscopy of strongly correlated phases in twisted trilayer graphene. *Nat. Mater.* **22**, 316–321 (2023).

3. Zondiner, U. *et al.* Cascade of phase transitions and Dirac revivals in magic-angle graphene. *Nature* **582**, 203–208 (2020).

4. Hao, Z. *et al.* Electric field–tunable superconductivity in alternating-twist magic-angle trilayer graphene. *Science* **371**, 1133–1138 (2021).

5. Saito, Y. *et al.* Isospin Pomeranchuk effect in twisted bilayer graphene. *Nature* **592**, 220–224 (2021).

6. Xie, M. & MacDonald, A. H. Weak-Field Hall Resistivity and Spin-Valley Flavor Symmetry Breaking in Magic-Angle Twisted Bilayer Graphene. *Phys. Rev. Lett.* **127**, 196401 (2021).

7. Moshe, M., Kogan, V. G. & Mints, R. G. Edge-type Josephson junctions in narrow thin-film strips. *Phys Rev B* **78**, 020510 (2008).

8. Chen, S. *et al.* Current induced hidden states in Josephson junctions. *Nat. Commun.* **15**, 8059 (2024).

9. Fei, Z. *et al.* Two-dimensional itinerant ferromagnetism in atomically thin Fe3GeTe2. *Nat. Mater.* **17**, 778–782 (2018).

10. Li, Y. & Baberschke, K. Dimensional crossover in ultrathin Ni(111) films on W(110). *Phys. Rev. Lett.* **68**, 1208–1211 (1992).
